# *NODULE INCEPTION* Recruits the Lateral Root Developmental Program for Symbiotic Nodule Organogenesis in *Medicago truncatula*

**DOI:** 10.1016/j.cub.2019.09.005

**Published:** 2019-11-04

**Authors:** Katharina Schiessl, Jodi L.S. Lilley, Tak Lee, Ioannis Tamvakis, Wouter Kohlen, Paul C. Bailey, Aaron Thomas, Jakub Luptak, Karunakaran Ramakrishnan, Matthew D. Carpenter, Kirankumar S. Mysore, Jiangqi Wen, Sebastian Ahnert, Veronica A. Grieneisen, Giles E.D. Oldroyd

**Affiliations:** 1 Sainsbury Laboratory, Cambridge University, Bateman Street, Cambridge CB2 1LR, UK; 2 Department of Cell and Developmental Biology, John Innes Centre, Norwich Research Park, Colney Lane, Norwich NR4 7UH, UK; 3 Laboratory for Molecular Biology, Wageningen University & Research, Droevendaalsesteeg 1, 6708 PB Wageningen, the Netherlands; 4 Earlham Institute, Norwich Research Park, Colney Lane, Norwich NR4 7UH, UK; 5 Noble Research Institute, 2510 Sam Noble Parkway, Ardmore, OK 73401, USA

**Keywords:** nitrogen, endosymbiosis, rhizobia, lateral root/nodule organogenesis, NODULE INCEPTION, CYTOKININ RESPONSE FACTOR, LATERAL ORGAN BOUNDARIES DOMAIN, YUCCA, auxin, *Medicago truncatula*

## Abstract

To overcome nitrogen deficiencies in the soil, legumes enter symbioses with rhizobial bacteria that convert atmospheric nitrogen into ammonium. Rhizobia are accommodated as endosymbionts within lateral root organs called nodules that initiate from the inner layers of *Medicago truncatula* roots in response to rhizobial perception. In contrast, lateral roots emerge from predefined founder cells as an adaptive response to environmental stimuli, including water and nutrient availability. *CYTOKININ RESPONSE 1* (*CRE1*)-mediated signaling in the pericycle and in the cortex is necessary and sufficient for nodulation, whereas cytokinin is antagonistic to lateral root development, with *cre1* showing increased lateral root emergence and decreased nodulation. To better understand the relatedness between nodule and lateral root development, we undertook a comparative analysis of these two root developmental programs. Here, we demonstrate that despite differential induction, lateral roots and nodules share overlapping developmental programs, with mutants in *LOB-DOMAIN PROTEIN 16* (*LBD16*) showing equivalent defects in nodule and lateral root initiation. The cytokinin-inducible transcription factor *NODULE INCEPTION* (*NIN*) allows induction of this program during nodulation through activation of *LBD16* that promotes auxin biosynthesis via transcriptional induction of *STYLISH* (*STY*) and *YUCCAs* (*YUC*). We conclude that cytokinin facilitates local auxin accumulation through *NIN* promotion of *LBD16*, which activates a nodule developmental program overlapping with that induced during lateral root initiation.

## Highlights

• Lateral roots and nodules show extensive overlap in organogenesis and transcription
• Nodule and lateral root initiation converges at an auxin maximum and *LBD16*
• Cytokinin promotes auxin biosynthesis via *NIN* and *LBD16* during nodule initiation

## Graphical Abstract

**Figure:**
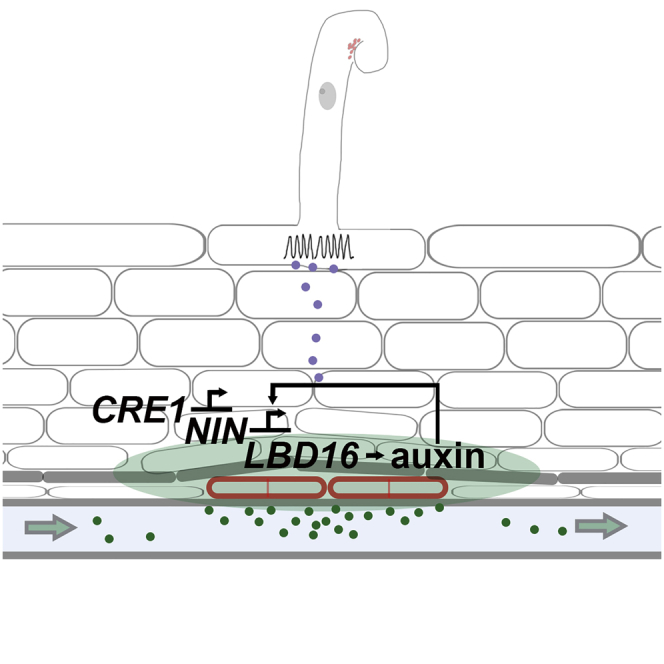

## Teaser

To overcome growth-limiting nitrogen deficiency in soils, legumes host nitrogen-fixing bacteria in symbiotic root nodules. Schiessl et al. show that despite different environmental stimuli and organ function, nodule and lateral root initiation converges on a local accumulation of the plant hormone auxin and a set of auxin-responsive regulators.

## Introduction

Nodules initiate as lateral root organs in response to the perception of rhizobial bacteria at the root surface. Rhizobial nodulation (Nod) factors activate symbiosis signaling in root epidermal cells, which in turn activates cytokinin signaling in the root cortex and pericycle [1, 2, 3, 4]. *CYTOKININ RESPONSE 1* (*CRE1*)-mediated signaling in inner-root tissues leads to the induction of the symbiosis-specific transcription factor *NODULE INCEPTION* (*NIN*), and both *CRE1* and *NIN* are indispensable for nodule initiation [5, 6, 7, 8, 9, 10]. While cytokinin signaling is both necessary and sufficient for the induction of nodules [7, 8], cytokinin suppresses lateral root development [11, 12, 13, 14], with *cre1* mutants showing increased lateral root emergence and decreased nodulation [13, 15].

Nodules and lateral roots initiate from pericycle, endodermal, and inner-cortical cells as a function of local auxin accumulation [16, 17]. Accompanying both lateral root and nodule development is upregulation of auxin-responsive *WOX5* and *PLETHORAs* at the initiation site of both organs [18, 19]. While lateral roots form from founder cells that are proposed to be primed by periodically oscillating auxin maxima, there is no evidence that nodules originate from such predefined founder cells [17, 20, 21]. Rather, the initiation of an auxin maxima during nodulation has been proposed to result from suppression of rootward polar auxin transport below the site of rhizobial recognition as a function of cytokinin recognition by *CRE1* [3, 22, 23, 24]. Pharmacological suppression of auxin transport promotes nodule organogenesis [22, 25], while genetic suppression of auxin transporters blocks nodule organogenesis [26, 27]. This work, together with computational modeling [28], has led to the proposition that symbiotic induction of cytokinin signaling blocks polar auxin transport below the site of rhizobial recognition, leading to a localized auxin maximum that coordinates nodule organogenesis.

These previous studies indicate that a number of parallels can be drawn between nodule and lateral root development, but their modes of initiation differ significantly. In this study, we directly compared lateral root and nodule development with high spatial and temporal resolution to identify the commonalities and differences that underlie their development. We demonstrate that lateral roots and nodules share overlapping developmental programs that converge on the formation and interpretation of an auxin maximum. This is exemplified by our finding that auxin-responsive *LOB-DOMAIN PROTEIN 16* (*LBD16*) is required for formation of both nodule and lateral root primordia. *NIN* and *LBD16* are necessary for cytokinin promotion of the auxin biosynthesis regulators *STYLISH* (*STY*) and *YUCCAs* (*YUC*), suggesting that the recruitment of *NIN* and *LBD16* into a symbiotic response allows cytokinin promotion of a root developmental program during nodulation.

## Results

### Lateral Roots and Nodules Show Extensive Overlap in Development and Transcription

**Figure 1:**
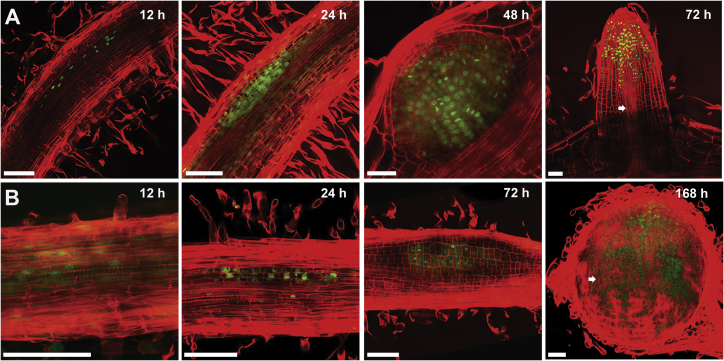
Lateral Roots and Nodules Show Overlapping Development (A and B) (A) Optical sections of lateral roots and (B) nodules hours (h) post induction. Red propidium iodide demarks cell walls and green EdU-labeled nuclei DNA replication. Arrowheads indicate vascular strands that in lateral roots are apparent by 72 hpi compared to nodules at 120–168 hpi. Scale bars:100 μm. See also Figure S1.

To better understand the commonalities and differences in lateral root and nodule development, we compared their organogenesis and correlated this with changes in gene expression. To initiate lateral roots in *Medicago truncatula*, we turned 2-day-old seedlings 135° to create a bend in the root [29], while nodules were induced with droplets of *Sinorhizobium meliloti* culture applied on the root susceptibility zone (Figures S1A and S1B). For both organs, we observed initial cell-cycle activation 12 h post induction (hpi) using the fluorescently labeled nucleotide analog 5-ethynyl-2-deoxyuridine (EdU). Anticlinal cell divisions initiated at 16 hpi and by 24 hpi primordia of both organs consisted of several cell layers, implying multiple rounds of periclinal cell divisions (Figures 1A and 1B). Lateral root primordia developed consistently faster than nodules, with cone shaped primordia at 48 hpi and fully emerged lateral roots at 72 hpi, whereas nodules developed as flat and concealed primordia up to 72 hpi, with nodule emergence occurring between 120–168 hpi (Figures 1A and 1B).

**Figure 2:**
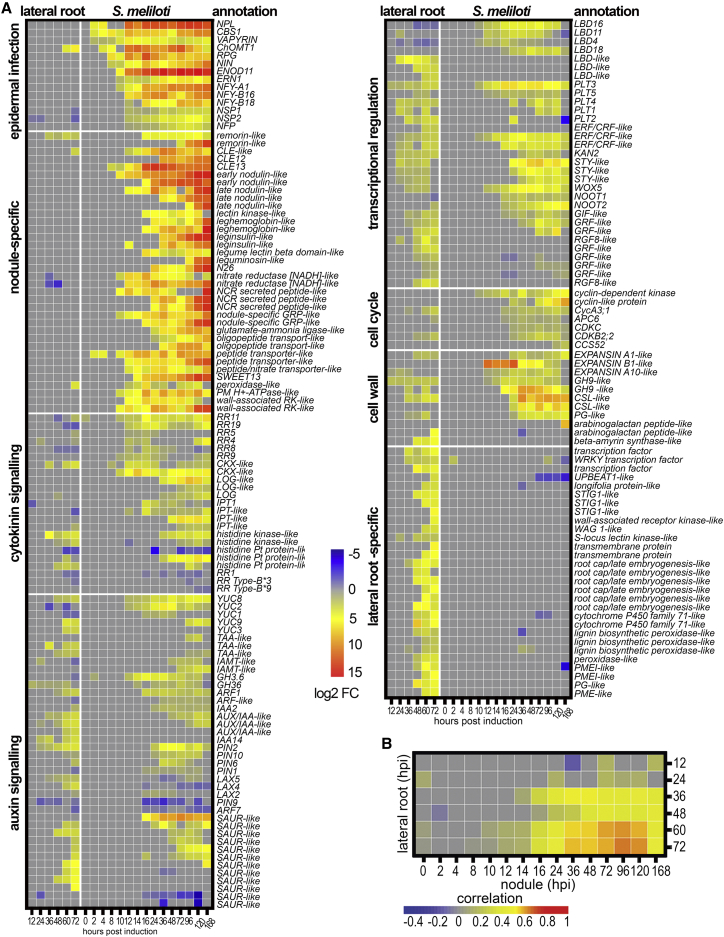
Lateral Roots and Nodules Show Overlap in Gene Expression (A) Heatmap showing selected genes induced during lateral root and nodule development with fold changes ≥±1.5; p < 0.05. Expression depicts log_2_ fold changes. (B) Correlation heatmap depicting the overlap between genes differentially expressed during lateral root and nodule organogenesis over a time course of development. See also Figures S1 and S2 and Data S1.

To correlate these developmental processes with gene expression changes, we performed RNA sequencing (RNA-seq) on 2- to 3-mm segments of gravitropically stimulated and non-stimulated roots at six time points between 12 and 72 hpi and of *S. meliloti* or mock spot-inoculated roots at 15 time points from 0 to 168 hpi (Figures 2A, and S1D–S1F). This revealed a high overlap in gene expression changes: 74% of upregulated genes and 81% of downregulated genes in lateral root development were similarly responsive in nodulation (Figures 2A, 2B, and S2; Data S1).

The earliest-responding genes to *S. meliloti* inoculation at 2 hpi were genes previously described as activated in the root epidermis [30]: *NPL*, *CBS1*, *VAPYRIN*, *ChOMT*, *RPG*, *NIN*, and *NF-YA1* (Figures 2A and S2; Data S1). Reporters for cytokinin signaling were induced with the onset of cell-cycle activation in the root cortex and pericycle at 10–12 hpi, and these showed little or no response during lateral root initiation (Figures 2A and S2; Data S1). Genes associated with auxin biosynthesis and signaling, together with a set of auxin-responsive transcriptional regulators, were among the earliest-upregulated genes with overlapping expression during lateral root and nodule development: *YUC*, *PLETHORAs (PLT)*, and *STY*, previously shown to regulate auxin metabolism, transport, and signaling during lateral root development in *Arabidopsis* [31, 32, 33, 34]. The upregulation of auxin-related genes coincides with the first significant auxin responses that occurred 12 h post *S. meliloti* inoculation, as evidenced by the auxin-response reporter *DIRECT REPEAT5* driving green fluorescent protein (*DR5-GFP*) (Figure S1C).

### *LBD16* Represents a Point of Convergence between Nodule and Lateral Root Development

**Figure 3:**
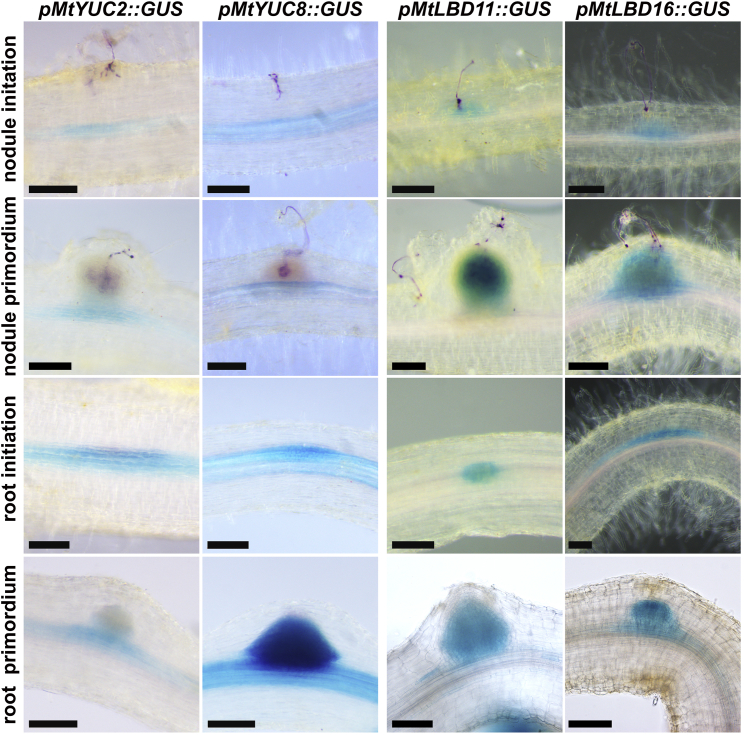
*LATERAL ORGAN BOUNDARIES* and *YUCCA*s Are Expressed during Root and Nodule Primordium Initiation and Development Expression patterns of *YUC2*, *YUC8*, *LBD11*, and *LBD16* during nodule and lateral root development visualized by GUS staining (blue). Rhizobial-expressed *LacZ* is stained magenta. Scale bars: 100 μm. See also Figure S3 and Data S2A and S2B.

During lateral root initiation in *Arabidopsis*, *YUC* and *LBDs* are activated and associated with the regulation and response to auxin [35, 36]. In our transcriptional profiling, we found *YUC2*, *YUC8*, *LBD11*, and *LBD16* induction within 12 h in response to rhizobial treatment, which was coordinated with the overall auxin response (Figures 2A, S1C, S2, and S3A; Data S2A and S2B). Promoter-β-glucuronidase (GUS) analysis of these four genes revealed that all were induced at the sites of lateral root and nodule initiation very early during primordia development (Figure 3).

**Figure 4:**
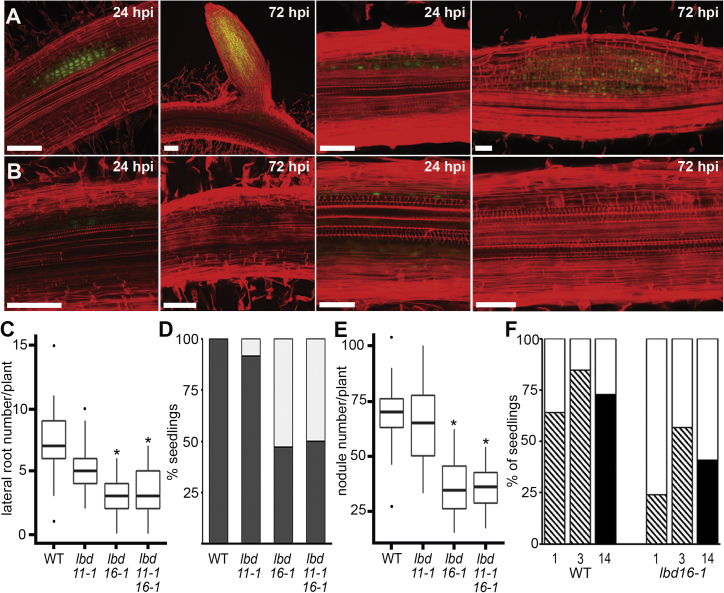
Lateral Root and Nodule Number Are Reduced in *lbd16* (A and B) Optical sections of lateral roots and nodules in (A) wild-type (WT) and (B) *lbd16-1* at 24 or 72 hpi. Scale bars: 50 μm. (C) Lateral root number in 14-day-old seedlings. Boxplots show median (thick line), second to third quartiles (box), minimum and maximum ranges (lines), and outliers (single points). A one-way Kruskal-Wallis rank-sum test showed that lateral root number is dependent on genotype; asterisks indicate significantly different (95% confidence) means compared with WT. n = 56 (WT), 58 (*lbd11-1*), 64 (*lbd16-1*), and 66 (*lbd11lbd16*). (D) Percentage of gravi-stimulated seedlings with ≥1 lateral roots (dark gray) or 0 lateral roots (white) in the bend 5 dpi in WT (n = 25), *lbd11-1* (n = 60), *lbd16-1* (n = 53), and *lbd11lbd16* (n = 60) showing significant reduction in the number of emerging lateral roots in *lbd16-1* and *lbd11lbd16* compared to WT. p = 0.166, 7.401e−07, and 1.413e−06 respectively; Fisher’s exact test. (E) Nodule number 21 days post *S. meliloti* inoculation. n = 13 (WT), 15 (*lbd11-1*), 14 (*lbd16-1*), and 14 (*lbd11lbd16*). A normal distribution allowed a one-way ANOVA test revealing the mean number of nodules differed significantly between genotypes; asterisks indicate significantly different (95% confidence) means compared to WT. (F) Percentage of seedlings with ≥1 primordia (hashed), ≥1 emerged nodule (black), or no structure (white) developing at the spot inoculation site at 1, 3, and 14 days post inoculation (dpi; n = 116 WT and 142 *lbd16-1*). *lbd16-1* showed significantly different rates of initiation of primordia or nodules at all time points: p = 0.003 (1 dpi), 0.022 (3 dpi), and 2.084e−04 (14 dpi); Fisher’s exact test. See also Figures S3 and S4 and Data S2A.

**Figure 5:**
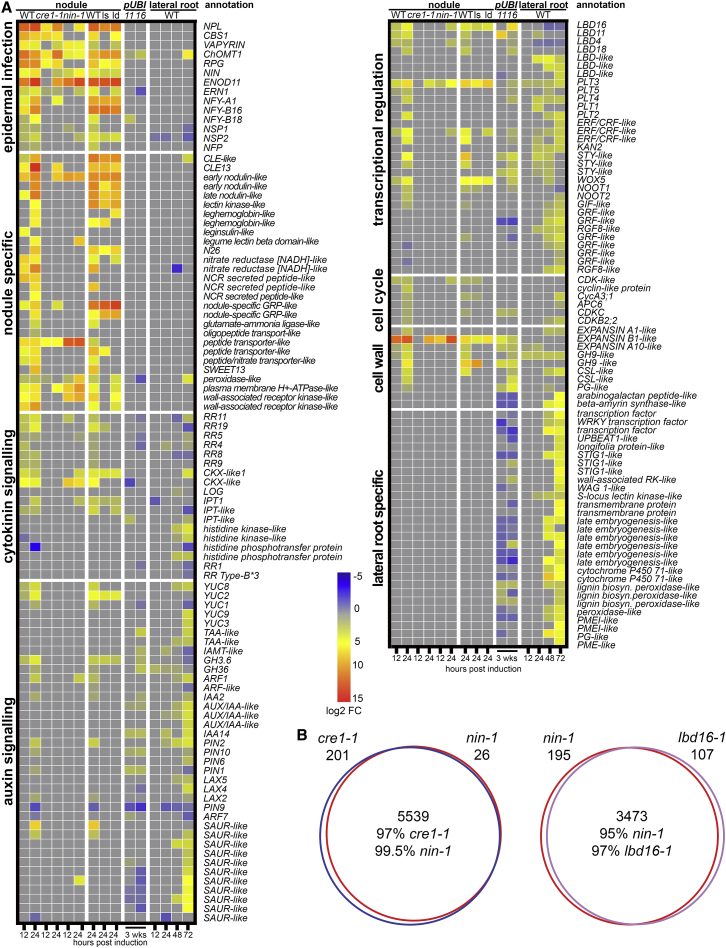
The Impact of *CRE1*, *NIN*, and *LBD16* on Nodulation-Associated Gene Expression (A) Heatmap of selected genes (as in Figure 2; see also Figure S2 for gene identifiers and Data S1) in WT (jemalong), *cre1-1*, and *nin-1* root sections at 12 and 24 h and WT (R108), *lbd16-1* (*ls*), and *lbd11lbd16* (*ld*) at 24 h post *S. meliloti* spot inoculation; response to *LBD11* (*11*) and *LBD16* (*16*) overexpression in 3-week-old hairy roots compared to control roots and during lateral root induction. Expression represents log_2_ fold changes. (B) Pairwise comparisons of all differentially expressed genes dependent on *cre1-1* (blue), *nin-1* (red), and *lbd16-1* (purple). *cre1-1 and nin-1* comparisons were to WT jemalong and *lbd16-1* to WT R108. See also Figures 2, S2, and S5 and Data S1.

To further assess the importance of *LBDs*, we identified mutants in *LBD11* (*lbd11-1*) and *LBD16* (*lbd16-1, lbd16-2*) (Figures S3B–S3G). Mutants in *lbd16* and *lbd11lbd16* showed significant defects in root organogenesis, with 50% reductions in the number of emerged lateral roots and nodules (Figures 4C–4F, S3D–S3G, and S4). This reduction is associated with early defects in cell division during primordium formation. In the most severe cases, root sections with no primordia emerging 72 hpi appeared to have terminated development after a few divisions (Figures 4A and 4B). From this, we infer that *LBD16* represents a convergence point between lateral root and nodule development, being important for the promotion of cell proliferation during organ initiation and early primordium development. Consistent with an early role in primordium formation, *lbd16* and *lbd11lbd16* mutants showed dramatic reductions in nodule-associated gene expression: 93% of rhizobial-induced genes at 24 hpi are *LBD16* dependent (Figures 5A, 5B, and S2; Data S1). This includes auxin-signaling genes, auxin regulators such as *STYLISH* and *YUCCAs*, and cell-wall-remodeling genes, all of which have overlapping expression in nodules and lateral roots.

### Overexpression of *LBD16* or *YUC2* Is Sufficient to Promote Root Primordia Formation

**Figure 6:**
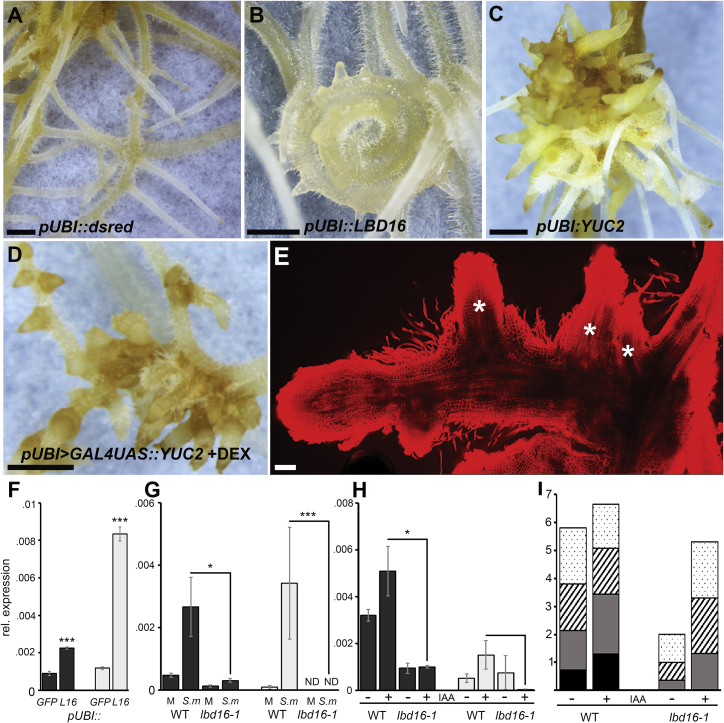
Overexpression of *LBD16* or *YUC2* Is Sufficient to Promote Root Primordia Formation (A–E) Constitutive expression of (A) *dsred* (control), (B) *LBD16*, or (C) *YUC2* under control of the *LjUBI* promoter in hairy roots. 44 out of 103 and 53 out of 57 transformed plants expressing *pLjUBI:LBD16* and *pLjUBI:YUC2*, respectively, showed similar phenotypes as depicted in (B) and (C). (D and E) (D) Bright field image and (E) optical sections of propidium iodide-stained root structures expressing dexamethasone (Dex)-inducible *YUC2* under control of the *LjUBI* promoter (*pLjUBI > GAL4UAS::MtYUC2*). 82 out of 167 plants transformed with *pLjUBI > GAL4UAS::MtYUC2* showed ectopic primordium induction 2 weeks post Dex treatment as depicted in (D) and (E) and Figures S5C–S5G compared to 3 out of 147 Dex treated plants transformed with *pLjUBI > GAL4UAS::GFP.* Ectopic primordia in (E) are indicated with asterisks. Scale bars: (A–D) 1 mm and (E) 50 μm. (F–H). (F) Quantification of transcript levels by qRT-PCR of STY-like (*Medtr1g023320*) (dark gray bars) and STY-like (*Medtr8g076620*) (light gray bars) in hairy roots constitutively expressing *LBD16* or *GFP* (*pUBI::LBD16* or *pUBI::GFP*), (G) *S. meliloti* and mock spot inoculation, and (H) IAA (+) and mock (−) treatment in WT and *lbd16-1* root sections at 24 hpi. Expression levels were measured by qRT-PCR and normalized to *HH3*. Statistical comparisons were performed as indicated. Values are the mean of 3 biological replicates ± SEM (Student’s t test; asterisks indicate statistical significance; ^∗^p < 0.05, ^∗∗^p < 0.01, ^∗∗∗^p < 0.001). (I) Number of lateral root primordia in 3-day-old WT and *lbd16-1* seedlings 24 h post IAA or mock treatment represented as means (n ≥ 11). The different stages of primordia development are indicated: dotted, stages I–II; striped, stage III; light gray, stages IV–V; black, emerged. Mock-treated *lbd16-1* seedlings had significantly fewer primordia compared to WT (Student’s t test; p < 0.001). IAA treatment significantly increased primordia number in *lbd16-1* seedlings (Student’s t test; p < 0.001), but not in WT. More stages IV–V and emerged primordia developed in WT than in *lbd16-1* in both treatments (Student’s t test; p < 0.001). See also Figures S5 and S6.

*LBD16* in *Arabidopsis* is induced by a localized auxin maximum and controls initial cell divisions during lateral root formation [32, 36, 37]. Our *LBD16* mutants in *M. truncatula* imply a similar function in this species. To further understand the role that *LBD16* plays during root organogenesis, we overexpressed *LBD16* in *M. truncatula* using the *Lotus japonicus UBIQUITIN1* (*LjUBI*) promoter. Roots overexpressing *LBD16* showed extensive curling with ectopic root primordia initiation (Figures 6A, 6B, S5A, and S5B). *LBD16* overexpression was associated with the constitutive induction of two STY-like transcription factors and these same *STY* genes were dependent on *LBD16* for their induction by rhizobia (Figures 5A, 6F, 6G, S2, and S5K). In *Arabidopsis*, *STY* control the regulation of the *YUC* auxin biosynthesis genes [33]. Expression of *YUC2* from the constitutive *LjUBI* promoter or induced through dexamethasone regulation resulted in initiation and emergence of ectopic root primordia (Figures 6C–6E, and S5C–S5G). From this, we conclude that *LBD16*, most likely through the regulation of auxin biosynthesis via *STY*, controls root organ initiation. However, the overexpression of *LBD16* led primarily to the expression of auxin-associated genes, but not the induction of nodule or lateral root-specific genes (Figure 5A). We conclude that induction of *LBD16* or promotion of auxin biosynthesis is sufficient to activate root primordia formation, but additional factors need to be coordinately induced to give rise to nodule or lateral root identity.

Similar to what has been reported in *Arabidopsis* [36], we found that indole-3-acetic acid (IAA) treatment of *M. truncatula* roots activates *LBD16* expression (Figure S5H), revealing that *LBD16* is responsive to auxin accumulation. IAA treatment of *lbd16-1* partially rescued the lateral root phenotype, enhancing the number of root primordia that formed. However, primordia emergence was still defective in *lbd16-1* (Figure 6I). Despite this partial rescue of the mutant phenotype by IAA treatment, we observed that the induction by both rhizobia and IAA of *STY*, *PLT3*, and a cell-wall-modifying POLYGALACTURONASE-like gene was abolished in *lbd16-1* (Figures 6H, S5I, and S5J). We conclude that *LBD16* is both responsive to auxin and responsible for the promotion of auxin, implying that *LBD16* has a function in the amplification of auxin accumulation.

### Cytokinin Promotion of *STY* and *YUC* Is a Function of *NIN* and *LBD16*

Induction of nodules in response to rhizobial bacteria is dependent on the cytokinin receptor *CRE1* and the cytokinin-inducible transcription factor *NIN* [4, 5, 9, 13]. Consistent with this, RNA-seq on *S. meliloti* spot-inoculated root segments of the *cre1-1* or *nin-1* mutants at 24 hpi revealed that 98.7% and 95.7% of *S. meliloti*-responsive genes are dependent on *CRE1* or *NIN*, respectively. Genes dependent on *LBD16* showed almost complete overlap with *CRE1* and *NIN* dependencies with the exception of infection-associated genes in the root epidermis and some nodule-specific genes (Figures 5A, 5B, and S2). Such a large overlap between *NIN*- and *LBD16*-dependent gene expression suggested the possibility that *LBD16* may function downstream of *NIN* and may be required for *NIN* induction of nodule initiation. Consistent with such a hypothesis, we observed that overexpression of *NIN* is sufficient to activate *LBD16* expression along with its known target *NF-YA1* [10] (Figure S5L).

**Figure 7:**
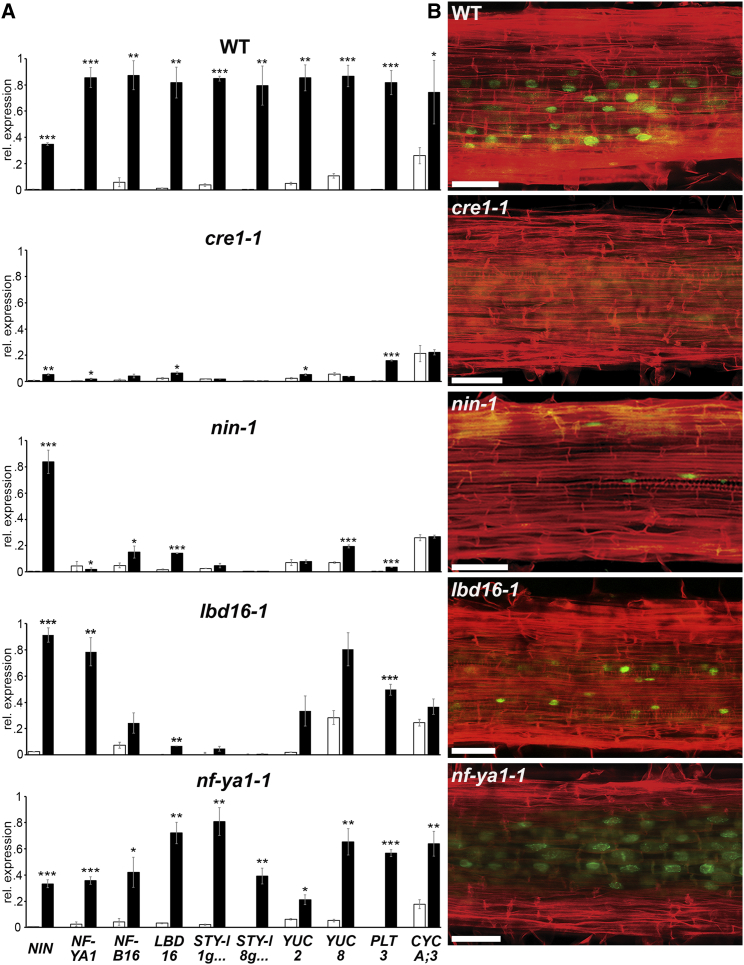
*NIN* and *LBD16* Mediate Auxin Regulators and Cell-Cycle Activation in Response to Cytokinin (A) Expression profiling on root segments treated with 100 nM (6-Benzylaminopurine) BAP for 24 h by qRT-PCR normalized to *HH3*. Statistical comparisons were performed between mock (white bars) and BAP (black bars). Values are the mean ΔCt values of three biological replicates normalized to the maximum value obtained for that gene within the ecotype. Data are presented ± SEM (Student’s t test; ^∗^p < 0.05; ^∗∗^p < 0.01, ^∗∗∗^p < 0.001). (B) Representative optical sections (≥20 roots analyzed) in root segments (susceptibility zone) treated with 100 nM BAP. Red, cell walls; green, cell-cycle activation. Scale bars: 50 μm. See also Figures S6A–S6C.

During nodulation, auxin accumulation and auxin signaling are activated by cytokinin at the site of rhizobial infection in a *CRE1*-dependent manner [22, 38], and we found that auxin accumulation in spot-inoculated root sections was *CRE1*- and *NIN*-dependent (Figure S6D). Wild-type *M. truncatula* roots respond to cytokinin treatments with upregulation of nodule-associated transcriptional regulators including *NIN*, *NF-YA1*, and *NF-YB16* and auxin-associated genes including *LBD16*, *PLT3*, *STY*, and *YUC* (Figures 7A and S6B). Furthermore, such cytokinin treatment is sufficient to promote cell-cycle activation in the root cortex, as evidenced by EdU staining (Figures 7B, S6A, and S6C). Such cytokinin responses are abolished in *cre-1-1* and *nin-1*, consistent with their role in cytokinin signaling and response. While cytokinin treatment of *lbd16-1* resulted in *NIN* and *NF-YA1* induction, we found that the induction of *STY* and *YUC* by cytokinin was significantly reduced in *lbd16-1*, as was the induction of *CYCLINA;3*. By contrast, loss of *NF-YA1-1* function appeared to have little effect on gene regulation and cell-cycle activation by cytokinin. We conclude that cytokinin promotion of auxin biosynthesis and cell division, which appears to uniquely occur in roots during nodulation, is the function of *NIN* and *LBD16.*

## Discussion

Our work demonstrates that lateral root and nodule development converge on a core root developmental program primarily associated with the formation and interpretation of a localized auxin maximum. The induction of this core developmental program is in part dependent on *LBD16*, which shows similar patterns of expression during nodule and lateral root development. *LBD16* has been shown to promote root organogenesis in response to an array of environmental stimuli, including hydropatterning and wounding [39, 40], and our work further supports the essential role that *LBD16* plays as a key integrator in the adaptation of root system architecture. We propose that the integration of *LBD16* into the symbiotic response to rhizobial bacteria is responsible for the recruitment of a core root developmental program and represents a point of convergence between nodule and lateral root development.

Nodulation is restricted to a group of plants in the so-called “nitrogen-fixing clade” and is specifically associated with the accommodation of nitrogen-fixing bacteria. A diversity of species within this clade show nodulation, and recent studies imply a single origin for the emergence of nodulation within this clade [41, 42]. Actinorhizal species (nodulating species outside the legumes) show nodules that possess a centralized vasculature [43], similar in morphology to lateral roots. Considering the new insights in nodule evolution, it seems likely that the unique architecture of the legume nodule is a derived state from the more primitive structures shown by actinorhizal plants. This is consistent with our studies that demonstrate a 75% overlap in the gene expression changes induced in lateral roots and nodules. We suggest that the initial stages of nodule evolution involved the recruitment of lateral root organogenesis into a symbiotic program, with later adaptations that created the unique features of the nodule.

The overlap between nodules and lateral roots appears to primarily converge on the response to an auxin maxima at the site of nodule and lateral root initiation. The auxin maxima during nodule formation was thought to be primarily caused by cytokinin and/or flavonoid-derived suppression of polar auxin transport [3, 22, 23]. However, here, we show that *YUC*s are activated below the site of the developing nodule primordia, and this precedes marker genes for auxin responses. We suggest that it is a combination of localized auxin biosynthesis, coupled with changes in polar auxin transport, that both contribute to the auxin maxima that forms in response to rhizobial recognition. Localized increases of intracellular auxin levels, mediated by *YUC*, have been proposed to promote further auxin accumulation in *Arabidopsis* leaves via reorientation of PIN-FORMED (PIN) proteins [44]. A similar situation could exist during nodulation whereby initial activation of local auxin biosynthesis below the site of rhizobial perception could modulate polar auxin transport to further promote auxin accumulation [24, 28].

Upregulation of *LBD16* upon rhizobial spot inoculation coincides with the formation of the auxin maxima and precedes activation of the cell cycle, implying an early role for *LBD16* in this process. Loss-of-function analysis showed that *LBD16* is necessary for the appropriate initiation of root and nodule primordia, and ectopic expression of *LBD16* showed promotion of root primordia and induction of many regulators of root development that are activated during both nodule and lateral root development. Previous work in *Arabidopsis* has shown that *LBD16* is required for the transition from root-founder cell identity to primordium cell identity, including activation of cell proliferation [40, 45, 46]. Expanding such observations into nodule initiation, we suggest that in the absence of founder cells, promotion of nodules involves activation of root organogenesis via cytokinin, which provides a novel route into the induction of *LBD16* that then promotes primordial cell identity and proliferation. Additional factors must also be induced that promote organ-specific differentiation into lateral roots or nodules.

While nodulating and non-nodulating species of plants respond to auxin treatment with emergence of nodule-like structures [47], only nodulating legumes do so in response to cytokinin [48]. Our work suggests that cytokinin promotion of *NIN* activates *LBD16* that then promotes local auxin biosynthesis through the induction of *STY* and *YUC*. This suggests that modifications to the *NIN*, *LBD16* regulon in legumes allowed a novel root developmental response to cytokinin that is critical for the promotion of nitrogen-fixing nodules. In *L. japonicus*, two *STY* genes are induced during nodulation in a manner dependent on *NF-YA1* [49], a direct target of *NIN.* However, in our work, we see *LBD16* dependence for cytokinin activation of *STY*, but not a dependence on *NF-YA1.* NIN-like proteins function in the adaptive response of roots to nitrogen availability [50, 51], and it is striking that *NIN* has been specifically recruited during the evolution of nodulation with no role in promoting lateral root development (Figure S6E) [41, 42]. The high degree of overlap in nodule and lateral root development implies that engineering efforts to transfer nodule organogenesis to species that lack this trait should focus on the cytokinin induction of root organogenesis, as well as on the functional characterization of components that specifically promote nodule organ identity, rather than the necessity to engineer the entire nodulation developmental program.

## STAR★Methods

### Key Resources Table

**Table:** 

REAGENT or RESOURCE	SOURCE	IDENTIFIER
**Bacterial and Virus Strains**		

*Sinorhizobium meliloti* strain *2011*	[52]	N/A
*Sinorhizobium meliloti* strain *2011 pXLGD4 lacZ strain*	[52]	N/A
*Agrobacterium rhizogenes* strain *AR1193*	[53]	N/A

**Chemicals, Peptides, and Recombinant Proteins**		

buffered nodulation media (BMN)	[54]	N/A
AVG-Cl (Aminoethoxyvinyl glycine hydrochloride)	ABCAM	ab145382, CAS: 55720-26-8
Luteolin	Sigma-Aldrich	L9283,CAS: 491-70-3
Terragreen	Oil-DriCompany	72111537
RNeasy Micro Kit	QIAGEN	Cat 74004
RNase free DNase Kit	QIAGEN	Cat 79254
Indole-3-acetic acid	Sigma-Aldrich	I5148, CAS: 6505-45-9
5-Bromo-4-chloro-3-indolyl-β-D-glucuronic acid, sodium salt trihydrate	Melford Laboratories	CAS:12954-41-9
Magenta-5-Bromo-6-chloro-3-indolyl-β-D-galactopyranoside (magenta-x-gal)	Melford Laboratories	CAS: 93863-88-8
Propidium iodide	Sigma-Aldrich	P4170, CAS:25535-16-4
5-ethynyl-2’-deoxyuridine (EdU)	Invitrogen	A10044
Alexa Fluor™ 488 5-Carboxamido-(6-Azidohexanyl), Bis(Triethylammonium Salt)), 5-isomer	Invitrogen	A10266
α-Amylase from Bacillus licheniformis	Sigma-Aldrich	A3403, CAS: 9000-85-5
RNA Transcriptor First Strand cDNA Synthesis Kit	Roche Diagnostics	04379012001
LightCycler 480 SYBR green I master	Roche Diagnostics	04707516001
6-Benzylaminopurine	Sigma-Aldrich	B3408, CAS:1214-39-7
Dexamethasone	Sigma-Aldrich	D4902, CAS:50-02-2

**Critical Commercial Assays**		

IAA and iP quantification by liquid chromatography-tandem mass spectrometry	[55, 56]	N/A
Illumina TruSeq Stranded mRNA HT kit	Illumina, performed by IMGM Laboratories	20020594
*BsaI-HF*	New England BioLabs	R3535
*BpiI*	Thermo Fisher Scientific	ER1011
T4 DNA Ligase 2,000,000 units/mL	New England BioLabs	M0202T

**Deposited Data**		

Short read sequencing data	this manuscript	GEO: GSE133612

**Experimental Models: Organisms/Strains**		

Medicago truncatula cultivar jemalong	Heritage Seeds Pty, Adelaide, AU	jemalong
Medicago truncatula ecotype R108 incl tnt insertion lines *NF20768 (lbd16-1), NF15962 (lbd16-2), NF20919 (lbd11-1)* originally obtained from Nobel Research institute LLC, Ardmore, USA (Cheng et al., 2014)	this manuscript	*lbd16-1, lbd16-2, lbd11-1*
Medicago truncatula ecotype A17 mutant *cre1-1*	[3]	*cre1-1*
Medicago truncatula ecotype A17 mutant *nin-1*	[5]	*nin-1*
Medicago truncatula ecotype A17 mutant *nfya1-1*	[57]	*nfya1-1*

**Oligonucleotides**		

For qRTPCR and genotyping oligos see Table S1	N/A	N/A

**Recombinant DNA**		

Golden Gate Level 0, distributed via https://www.ensa.ac.uk	GeneArt, Thermo Fisher Scientific	N/A
Binary plasmids generated using Golden Gate Cloning [58], details see Table S2	this manuscript	N/A
Dex inducible system *(GVG and 6xGAL4UAS)*	[59]	N/A

**Software and Algorithms**		

Sequence info M. truncatula Mt4.0v1 genome retrieved Phytozome https://phytozome.jgi.doe.gov	[60, 61]	N/A
R package STAR	[62]	N/A
Feature Counts in R package Rsubread	[63]	N/A
R package edgeR	[64]	N/A
R package DESeq2	[65]	N/A
Synonym locus ID matches obtained from Uniprot	[66, 67]	N/A
fluorescence quantification software in FIJI	[68]	N/A
HMMER3.1b2 HMMSEARCH, HMMALIGN	http://hmmer.org/	N/A
Lotus genome annotation retrieved	ftp://ftp.kazusa.or.jp/pub/lotus/	N/A
Aligning sequences with MAFFTv72712	[69]	N/A
Extacting alignments with Jalview	[70]	N/A
Interactive Tree of Life Visualization Tool iToL	[71]	N/A

### Lead Contact and Materials Availability

Further information and requests for resources and reagents should be directed to and will be fulfilled by The Lead Contact, Giles E.D. Oldroyd (gedo2@cam.ac.uk), subject to material transfer agreements. This study did not generate unique reagents.

### Experimental Model and Subject Details

#### Plant material and S. meliloti strains including growth conditions

*Medicago truncatula* ecotypes jemalong, cultivar Jester, and ecotype R108 were used in this study. Jemalong was used to perform spot inoculation and hairy root transformations and as wild-type for comparisons to *cre-1*, *nin-1*, and *nf-ya1-1*, previously described [3, 5, 57]. All *Tnt1* retrotransposon insertion lines described (*NF20768 (lbd16-1)*, *NF15962 (lbd16-2)*, and *NF20919 (lbd11-1*) were derivatives of the R108 ecotype and obtained from the *Tnt1* Retrotransposon Mutant Collection (Noble Research Institute, Ardmore USA [72]; and as such R108 was used as the wild type for analysis of these mutants. Genotyping was performed using TntF and TntR oligos combined with the corresponding forward and reverse oligos encompassing the insertions (Table S1).

Seeds were scarified, surface sterilized with 10% (v/v) bleach solution, stratified for 3 days at 4°C and germinated on water agar plates. Plants were grown in sterile conditions in controlled environment rooms at 22°C (80% humidity, 16 h light/8 h dark, 300 μmol m2 s-1 light intensity) on filter paper-lined agar media in sealed plates unless otherwise specified. For spot inoculation seedlings were grown for 2 days on buffered nodulation medium (BNM) [54] supplemented with 1 μM aminoethoxyvinylglycine (AVG; Sigma-Aldrich Company Ltd, Darmstadt, Germany) at 22°C (16 h light/8 h dark, 300 μmol m-2 s-1 light intensity). *Sinorhizobium meliloti* strain *2011* [52] was grown in minimal medium supplemented with 3 μM luteolin (Sigma-Aldrich Company Ltd, Darmstadt, Germany) and diluted to a final concentration of 0.02 OD 600 nm using Fahraeus medium. The mock treatment consisted of Fahraeus medium with luteolin diluted to an equivalent concentration as the inoculum. Approximately 1 μL of *S. meliloti* suspension or mock treatment was inoculated onto the susceptibility zone (where the root hairs first appear) and marked by puncturing the filter paper alongside the site of inoculation. After periods ranging from 0 to 168 h, 2 mm sections of the root alongside the site of inoculation were harvested for RNA isolation or microscopy.

For spray inoculation of *S. meliloti* for plants grown on plates, seedlings were grown under similar conditions as described above. Roots of 1-day-old seedlings were covered with filter paper and sprayed with 2 mL *S. meliloti* of final concentration 0.02 OD 600 nm grown in minimal medium without luteolin. For inoculation of hairy roots, composite plants were transferred to terragreen:sharp sand mix (1:1) (Oil-DriCompany, Wisbech, UK) in P60 trays and left to grow for 7 days before inoculation with *S. meliloti 2011 pXLGD4 (lacZ)* (1.5 mL of overnight culture per plant diluted in liquid BNM to 0.02 OD 600 nm). Plants were grown for up to a further 4 weeks for nodule quantification and histochemical staining.

#### Bacterial strains

*Agrobacterium rhizogenes strain AR1193* was used to introduce all binary vectors used in this study (Table S2) to *M. truncatula* jemalong seedlings following a previously published transient hairy root transformation protocol [53].

### Method Details

#### Lateral root induction using gravi-stimulation

For lateral root induction [29], seedlings were grown for 2 days on modified Fahraeus medium plates and turned 135° for 12 h, then subsequently returned to their original orientation. Control plants were marked at the root tip at the time point of turning but left to grow straight. After periods ranging from 12 to 72 h, material was harvested for RNA isolation or microscopy or lateral roots were left to grow for 5 days before lateral root number was scored.

#### Construct production

The Golden Gate modular cloning system was used to prepare the plasmids [58]. This included a restriction digest-ligation protocol of 25 cycles of 3 min at 37°C and 4 min at 16°C using T4 DNA Ligase (New England BioLabs, Ipswich, UK) combined with the restriction enzymes *BsaI*-*HF* (New England BioLabs, Ipswich, UK) and *BpiI* (Thermo Fisher Scientific, Waltham, USA) for level1 and level2 assembly, respectively. All Level 0 s used in this study are held for distribution in the ENSA project core collection (https://www.ensa.ac.uk/) and are listed along with the binary plasmid details in Table S2. Sequences were domesticated, synthesized and cloned into pMS (GeneArt, Thermo Fisher Scientific, Waltham, USA). Sequence information for *Medtr7g096530 (LBD16)*, *Medtr4g060950 (LBD11)* and *Medtr6g086870 (YUC2)* and *Medtr7g099330 (YUC8)* were obtained from the *M. truncatula* Mt4.0v1 genome via Phytozome (https://phytozome.jgi.doe.gov) [60]. The Dex-inducible system (*GVG* and *6xGAL4UAS*) was adapted from the original system [59].

#### Hairy root transformation

Transformed *Agrobacterium* strains *AR1193* [53] harboring the binary vectors (Table S2) including the *AtUBI:dsred* selection marker were cultured on LB medium plates supplemented with the corresponding antibiotics for 36 h at 28°C. For transformation, *Agrobacteria* were washed off the plates and resuspended using 1 mL of distilled water. The bacterial suspension was used to dip 1-day old seedlings after the root tip had been cut off (1/4 of total root length). Dipped seedlings were subsequently transferred to and grown on modified Fahraeus medium plates for 3 weeks. Selection was performed using a Leica M205FA stereo microscope with LED illumination and filters for *dsred*. Images of hairy root structures were obtained using a Leica DFC310FX color camera (Leica Microsystems, Wetzlar, Germany).

#### Hormone and chemical treatments

Dexamethasone (Dex; Sigma-Aldrich Company Ltd, Darmstadt, Germany), indole-3-acetic acid (IAA; Sigma-Aldrich Company Ltd, Darmstadt, Germany) and luteolin were dissolved in 70% ethanol. AVG and 6-Benzylaminopurine (BAP; Sigma-Aldrich Company Ltd, Darmstadt, Germany) were dissolved in water. Mock treatments were equal volumes of each solvent in the agar media. For BAP plate treatments (100 nM) and IAA plate treatments (100 nM) 2-day old seedlings were grown on BNM plates for 24 h with either BAP or IAA and supplemented with 1 μM AVG to replicate spot inoculation conditions. For the lateral root primordium assay 2-day old seedlings were transferred to modified Fahraeus medium supplemented with IAA (100 nm) or mock for 24 h. For Dex treatments, 3-week old plants with transformed roots were transferred to BNM agar plates supplemented with KNO_3_ (potassium nitrate, 2.5 mM) and either Dex (1 μM) or mock treatment, with a piece of filter paper placed over the root systems to ensure full contact with the additives.

#### Hormone quantification by liquid chromatography-tandem mass spectrometry

For extraction of auxin from *M. truncatula* root material, ∼10 mg of snap-frozen spot-inoculated root material was used. Tissue was ground to a fine powder using 3-mm stainless steel beads at 50 Hz for one minute in a TissueLyser LT (QIAGEN, Germantown, USA). Ground root samples were extracted with 1 mL of cold methanol containing [phenyl 13C6]-IAA (0.1 nmol/mL) as an internal standard in a 2-mL eppendorf tube. The tubes were vortexed and sonicated for 10 min in a Branson 3510 ultrasonic bath (Branson Ultrasonics, Eemnes, Netherlands) and placed overnight in orbital shaker at 4°C. The samples were centrifuged for 10 min at 11,500 rpm in a Heraeus Fresco 17 centrifuge (Thermo Fisher Scientific, Waltham, USA) at 4°C, after which the organic phase was transferred to a 4-mL glass vial. The pellets were re-extracted with another 1 mL of cold methanol. The combined methanol fractions were pooled and MeOH evaporated in a speed vacuum system (SPD121P, Thermo Savant, Hastings, UK) at room temperature. Residues were resuspended in 1 mL milliQ (1% formic acid) and then loaded on a 30mg/1cc Oasis MCX cartridge (Waters Corporation, USA). The cartridge was equilibrated with 1 mL of MeOH and 1 mL milliQ (1% formic acid) prior to sample loading. Subsequently the cartridge was washed with 1 mL milliQ (1% formic acid) and eluted with 1 mL of 100% MeOH. The MeOH was evaporated in a speed vacuum (SPD121P, Thermo Savant, Hastings, UK) at room temperature and the residue resuspended in 100 μl acetonitrile:water:formic acid (30:70:0.1, v/v/v). The sample was filtered through a 0.45 μm Minisart SRP4 filter (Sartorius, Goettingen, Germany) and measured on the same day. Auxin was analyzed on a Waters Xevo TQs tandem quadruple mass spectrometer as previously described [55].

#### Gene expression analysis

For spot inoculation and lateral root induction time-course experiments, roots were dissected as 2- to 3-mm segments around the spot of inoculation or mock treatment. For BAP and IAA response experiments, segments were dissected around the susceptibility zone marked at the time of treatment. About 50 to 60 segments were pooled to obtain 1 biological replicate, with 3-6 biological replicates per treatment/genotype were analyzed. RNA was extracted using the RNeasy Micro Kit (QIAGEN, Germantown, USA) and the RNase free DNase kit (QIAGEN, Germantown, USA) was used to remove genomic DNA. For reverse transcription of 1 μg total RNA, Transcriptor First Strand cDNA Synthesis Kit was used according to the manufacturer’s instructions (Roche Diagnostics GmbH). Quantitative real-time polymerase chain reactions (qRT-PCR) were performed in technical triplicates in the LightCycler 480 System using LightCycler 480 SYBR green I master (04707516001, Roche Diagnostics GmbH, Mannheim, Germany) in a total reaction volume of 10 μl. The primer pairs used for gene expression analysis are listed in Table S1.

#### RNA-Seq

RNA sequencing (RNA-Seq) was performed by IMGM Laboratories (Martinsried, Germany). RNA-Seq libraries were prepared with the Illumina TruSeq Stranded mRNA HT kit and sequencing of the libraries was performed on the Illumina NextSeq500 next generation sequencing system using the high output mode with 1 × 75 bp single-end read and 2 x 150 bp paired-end read chemistry (Illumina, Cambridge, UK).

#### Histochemical assays and cellular stains

For GUS or X-Gal staining roots were washed in water and immediately fixed in 90% acetone on ice for 1 h. Subsequently, the acetone was replaced by a wash solution containing 50 mM phosphate buffer pH 7.2. The wash buffer was replaced by GUS staining buffer containing 50 mM phosphate buffer pH 7.2, 0.5 mM K3Fe(CN)6 (potassium ferricyanide), 0.5 mM K4Fe(CN)6 (potassium ferrocyanide) and 2 mM 5-bromo-4-chloro-3-indolyl-beta-D-glucuronide (X-Gluc, Melford Laboratories, Ipswich, UK), vacuum infiltrated for 15 min and incubated at 37°C overnight. For X-Gal staining, the tissue was washed in 50 mM phosphate buffer pH 7.2 and fixed in 2.5% glutaraldehyde by vacuum infiltration for 15 min and incubation at room temperature for 1 h. Tissue was washed 3X in Z-buffer containing 100 mM phosphate buffer pH 7, 10 mM KCl, 1 mM MgCl2 and incubated in X-Gal staining buffer (Z-buffer supplemented with 5 mM K3Fe(CN)6, 5 mM K4Fe(CN)6 and 0.08% Magenta-5-Bromo-6-chloro-3-indolyl-B-D-galactopyranoside; (X-Gal, Melford Laboratories, Ipswich, UK) at 28°C overnight and washed with water 3 times. Tissue was cleared, stored and imaged in chloralhydrate solution. Images were obtained using a Leica DM6000 compound microscope 20X air objective with bright field settings (Leica Microsystems, Wetzlar, Germany).

For combined 5-ethynyl-2-deoxyuridine (EdU; Invitrogen, Thermo Fisher Scientific, Waltham, USA) and modified pseudo-Schiff-propidium iodide (PI; Sigma-Aldrich Company, Darmstadt, Germany) staining, we modified the published method [56] for roots: root segments were transferred to growth medium supplemented with 10 μM EdU. At transfer, plants were covered with filter paper and sprayed with liquid BNM or modified Fahraeus medium supplemented with 20 μM EdU and incubated for 5 min before the filter paper was removed. After 4 additional h of growth on growth media supplemented with EdU, 1 cm root sections centered around the susceptibility zone were dissected and dehydrated for 15 min in an ethanol dilution series (15%, 30%, 50%, 70%, 85%, 95%, and 100% ethanol) and stored in 100% ethanol overnight. The samples were rehydrated through the same ethanol dilution series and incubated at 37°C overnight in 0.3 mg/mL alpha-amylase (Sigma-Aldrich Company, Darmstadt, Germany) in phosphate buffer (20 mM pH 7.0, 2 mM NaCl, 0.25 mM CaCl2). All Edu labeling and PI staining steps were performed at room temperature with gentle shaking. First, the root sections were rinsed in water and incubated in solution containing 10 mM Alexa 488-azide (Invitrogen, Thermo Fisher Scientific, Waltham, USA) and 100 mM Tris pH 8.5 for 1 h, followed by 30 min in solution containing 10 mM Alexa 488-azide, 100 mM Tris, 1 mM CuSO4, 100 mM ascorbic acid, pH 8.5. The roots were subsequently washed three times in water, treated in 1% periodic acid for 30 min, washed twice in water, and incubated in Schiff-PI reagent for 2 h. The samples were cleared with chloral hydrate solution (Sigma-Aldrich Company, Darmstadt, Germany) for 1 h and mounted in Hoyer’s medium [56]. Imaging was performed with a Zeiss 700 confocal scanning microscope with excitation at 488 nm and emission filters set to 572–625 nm for propidium iodide and 505–600 nm for EdU using a 20X air lens objective (Carl Zeiss AG, Oberkochen, Germany). Images were processed using FIJI [73].

#### *DR5::GFP* fluorescence quantification

Individual seedlings stably transformed with a *DIRECT REPEAT5* element driving nuclear localized green fluorescent protein (*DR5::GFP*), were visualized at the site of droplet application using the Leica M205FA stereo microscope with standard microscopy settings (Leica Microsystems, Wetzlar, Germany). The progression of each seedling was tracked by keeping them undisturbed inside the sealed growth plates, with plates removed from the growth chamber only at the visualization time points. The images were analyzed for mean fluorescence inside regions of interest (ROIs, squares with side length equal to the width of the root and centered at the droplet treatment) using FIJI software, similarly to previous studies [73, 74]. At each time point, we quantified the mean fluorescence inside the inoculation spot ROI, and two identically sized ROIs, positioned 3-root widths above and below the site of inoculation. The ratio of inside to average outside fluorescence was then calculated (ROI[spot]/ ((ROI[above]+ROI[below])/2).

#### Phylogenetic analysis

LATERAL ORGAN BOUNDARY (LOB) proteins were detected in the *Arabidopsis*, Medicago and Lotus proteomes using HMMER3.1b2 HMMSEARCH (hmmer.org). The inputs to this program were the Pfam hidden Markov model (HMM), DUF260 (PF00271, Pfam release 30), and the protein datasets from the *Arabidopsis* (Araport11), Medicago (Phytozome, V10) and Lotus genome annotations (ftp://ftp.kazusa.or.jp/pub/lotus/). The protein sequences detected were aligned back to the HMM using HMMER3.1b2 HMMALIGN. Gap columns in the alignment were removed, sequences with less than 70% coverage across the alignment were removed and the longest sequence for each gene from the set of splice versions was used for phylogenetic analysis. Phylogenetic analysis was carried out using the MPI version of RAxML v8.2.9 with the following method parameters set: -f a, -x 12345, -p 12345, -# 100, -m PROTCATJTT. The tree was mid-point rooted and visualized using the Interactive Tree of Life (iToL) tool [68]. Clades that included MtLBD11 and MtLBD16 were pruned from the tree and are displayed in Data S2A.

A similar procedure was used to identify YUCCA genes from the same genomes (Data S2B). The HMM FMO-like (PF00743, Pfam release 30) was used with HMMSEARCH to obtain the proteins. Most proteins showed insufficient coverage across the domain after aligning with HMMALIGN, however most of them aligned well at the N-terminal end of this model. Therefore a new HMM was built by aligning the full lengths of these sequences with MAFFT v7.2712 [71]. The conserved region of the alignment was extracted using Jalview [69] and used to build the new HMM with HMMER3.1b2 HMMBUILD. Then the same procedure as described above for the LOB family was repeated using the new HMM, except that sequences with less than 50% coverage across the alignment were removed before running RAxML.

### Quantification and Statistical Analysis

#### RNA-seq

Reads from the RNA-sequencing experiments provided as raw fastq data were quality controlled and mapped to the *M. truncatula* reference genome version 4.0 (Mt4.0v1) [70] using R package STAR [61]. The counts and RPKM (Reads per kilobase per million mapped reads) values were calculated with featureCounts in R package Rsubread [62]. Non-metric multidimensional scaling was exploited to account for outliers. At least 3 biological replicates were always included in the full analysis. Genes that showed low expression throughout all samples were removed by measuring CPM (counts per million) values using R package edgeR [63]. Differentially expressed genes (DEGs) were identified by pairwise comparisons of raw counts of mock treatment versus experimental treatment, using the R package DESeq2 [64] with the threshold of absolute fold change of over 1.5 and a false discovery rate (FDR) corrected p value more significant than 0.05. The heatmaps of differential expression were plotted with R package pheatmap. The synonyms for *M. truncatula* locus ids were obtained by manual curation and by retrieving names from the Uniprot database using BLAST matches [65, 66]. The descriptions for each genes were obtained from Phytozome (Data S1).

#### qRTPCR

Expression values of minimum three biological replicates in three technical replicates were analyzed using the Pfaffl method with histone H3 (HH3) as reference [67]. Statistical comparison was performed between WT and mutants or treatment and corresponding mock. Values depicted in bar charts are the mean of minimum 3 biological replicates ± SEM (Student’s t test; ^∗^ p < 0.05; ^∗∗^ p < 0.01, ^∗∗∗^ p < 0.001).

#### Phenotyping

Data on number of lateral roots and nodules was depicted in boxplots which show the median (thick line), second to third quartiles (box), minimum and maximum ranges (lines), and outliers (single points). Normal distribution of data was tested using the Shapiro-Wilk normality test. For pairwise comparisons statistical analysis was performed using either unpaired Student’s t test, Wilcoxon test or Fisher’s exact test. For multiple comparisons, one-way analysis of variance (one-way ANOVA) or one-way Kruskal-Wallis rank sum test, followed by Tukey multiple comparisons of means or Dunn test. The R statistical package was used for these analyses. Sample size n is provided in the figure legends and refers to the number of individual plants. Statistical tests and significance levels are provided in the figure legends.

### Data and Code Availability

The short-read sequencing data generated in this study have been deposited at the National Center for Biotechnology Information Gene Expression Omnibus, with accession number GEO: GSE133612. Lists of differentially expressed genes are compiled in Data S1.

## Supplemental Information

Document S1. Figures S1–S6 and Tables S1 and S2

Data S1. List of Differentially Expressed Genes Compiled from All Experiments, Related to Figures 2 and 5 and STAR Methods

Data S2. Phylogenetic Analysis, Related to Figures 3 and 4 and STAR Methods (A) Phylogenetic analysis of the *LBD* gene family. The tree shows a clade of 62 proteins out of 139 LBD-like proteins from *Arabidopsis thaliana, Lotus japonicus, and Medicago truncatula*. (B) Phylogenetic analysis of the *YUCCA* gene family. The tree shows all YUCCA-like proteins identified from *Arabidopsis thaliana, Lotus japonicus, and Medicago truncatula*. Genes functionally characterized in this study are labeled in red. Green dots indicate bootstrap values > = 90%. Related to Figures 3 and 4.

Document S2. Article plus Supplemental Information
