# Supplementary material for: NODULE INCEPTION Recruits the Lateral Root Developmental Program for Symbiotic Nodule Organogenesis in Medicago truncatula
Source: Curr Biol. 2019 Nov 4;29(21):3657–3668.e5. doi: 10.1016/j.cub.2019.09.005 (PMC6839406; doi:10.1016/j.cub.2019.09.005)
Supplement: Document S1. Figures S1–S6 and Tables S1 and S2 [file mmc1.pdf]

Current Biology, Volume *29*

Supplemental Information

NODULE INCEPTION Recruits the Lateral Root Developmental Program for Symbiotic Nodule Organogenesis in *Medicago truncatula*

Katharina Schiessl, Jodi L.S. Lilley, Tak Lee, Ioannis Tamvakis, Wouter Kohlen, Paul C. Bailey, Aaron Thomas, Jakub Luptak, Karunakaran Ramakrishnan, Matthew D. Carpenter, Kirankumar S. Mysore, Jiangqi Wen, Sebastian Ahnert, Veronica A. Grieneisen, and Giles E.D. Oldroyd

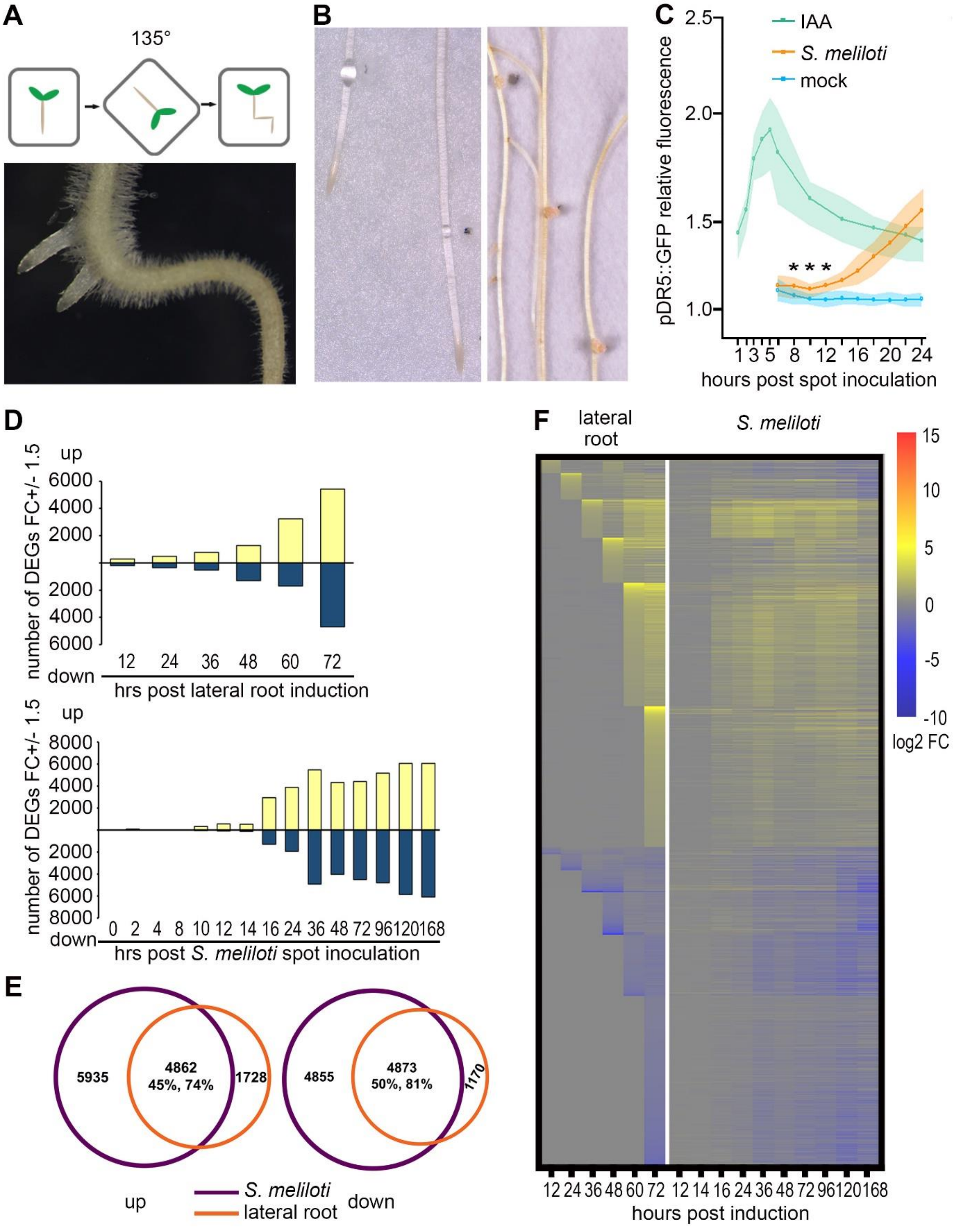

Figure S1. Induction of nodules and lateral roots. Related to Figures 1 and 2. (A) To initiate lateral roots we used a gravitropic stimulation assay. Lateral roots emerge from prebranch sites within and close to the induced bend at a frequency of > 95 %. **(B)** To induce nodules, droplets of *Sinorhizobium meliloti* suspension were placed on the susceptibility zone of the

root which coincides with the onset of the differentiation zone where the root hairs emerge (marked on the filter paper). Nodules initiated from these sites at a frequency of 85 % (n=53) **(C)** Auxin signalling response during nodulation measured with *pDR5::GFP-NLS* fluorescence intensity at the site of spot inoculation compared to neighbouring non-inoculated areas of the root. Spot treatments included *S. meliloti* (orange, n= 22), 2.5 µM IAA (green, n=18), and mock (blue, n=14). Shading indicates 25-75% quantiles and asterisks indicate significant differences between *S. meliloti* and mock treatment at the specific timepoints (Student's *t* test, * $P < 0.05$). All datapoints in *S. meliloti* treatments after 12 hpi are significantly different to the mock treatment with a $P < 0.001$. **(D)** Number of differentially expressed genes (DEGs) over a time course of lateral root induction and post *S. meliloti* spot inoculation. Yellow bars represent upregulated genes and blue bars represent downregulated genes. The resolution of these transcriptome datasets is dependent on the predictability of capturing cells undergoing nodule and lateral root development: while nodules formed precisely at the location of rhizobial inoculation, lateral roots could emerge anywhere within the root bend (Figure S1A) consistent with initiation from prebranch sites, as previously reported [S1]. Furthermore, no nodules formed in the absence of *S. meliloti* inoculation, but we cannot rule out that we captured prebranch sites and early lateral root primordia in unbent control root segments. For these reasons, we believe we have better resolution during nodule initiation than we have during lateral root initiation. **(E)** Overlap of DEGs between lateral root induction (orange circles) and post *S. meliloti* spot inoculation (purple circles) across all time points; 74% of upregulated DEGs and 81% of the downregulated DEGs during lateral root induction are shared with the DEGs post *S. meliloti* spot inoculation. **(F)** Heatmap of all differentially expressed genes in response to lateral root induction 12-72 hpi and in response to *S. meliloti* spot inoculation 12 – 168 hpi in wild type. To compare the development of lateral roots and nodules we sorted all differentially expressed genes of the lateral root dataset according to the timepoints of induction and compared this to expression during nodule initiation. Expression levels are depicted as log_2 fold changes, (also see Data S1).

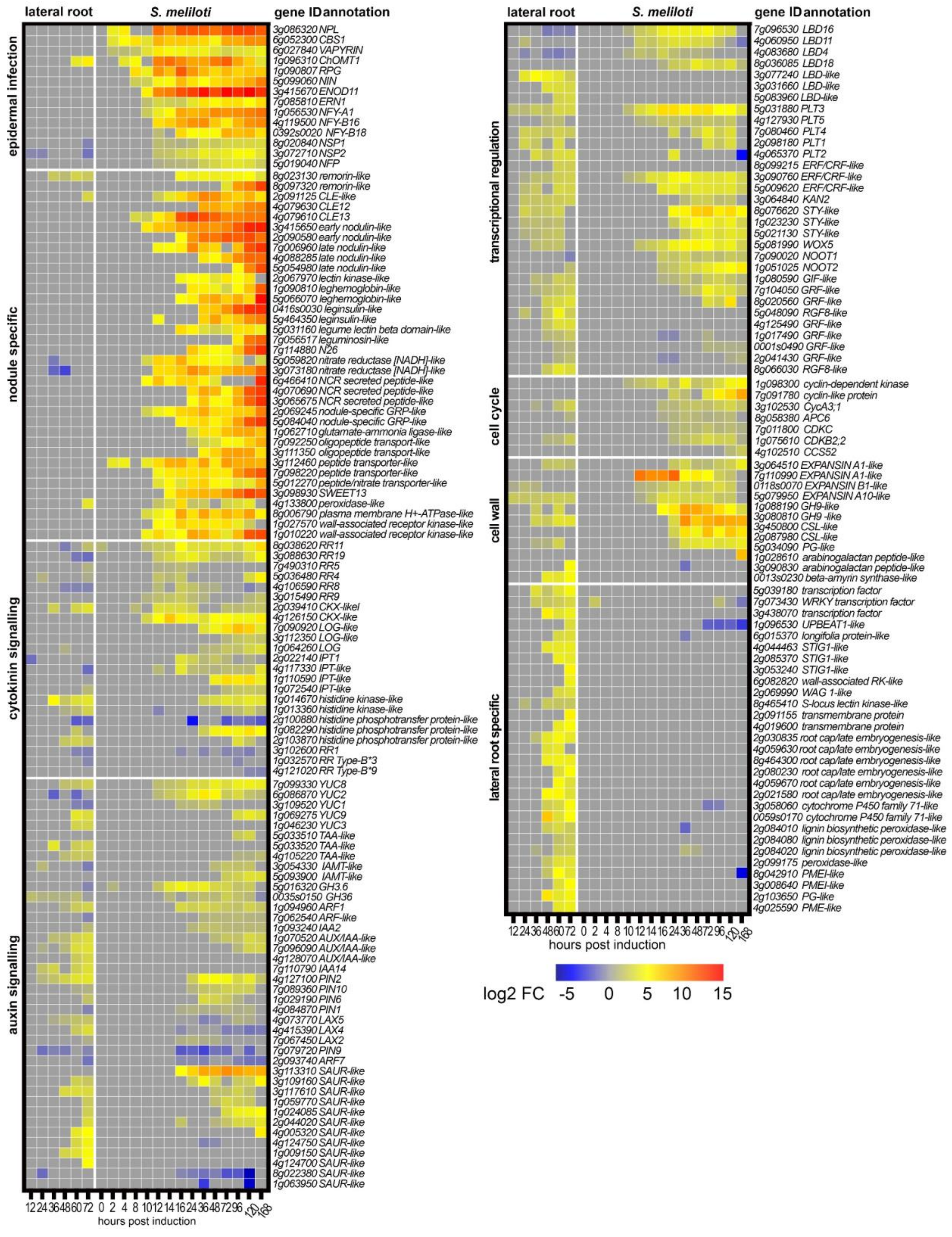

Figure S2. Lateral root and nodule initiation share genes involved in auxin signalling and transcriptional regulation. Related to Figures 2 and 5 and Figure S1. Heatmap of selected DEGs during lateral root and nodule induction in wild-type with gene identifiers as annotated in Mt4.0v1 included for all genes. Expression levels are depicted as log_2 fold changes.

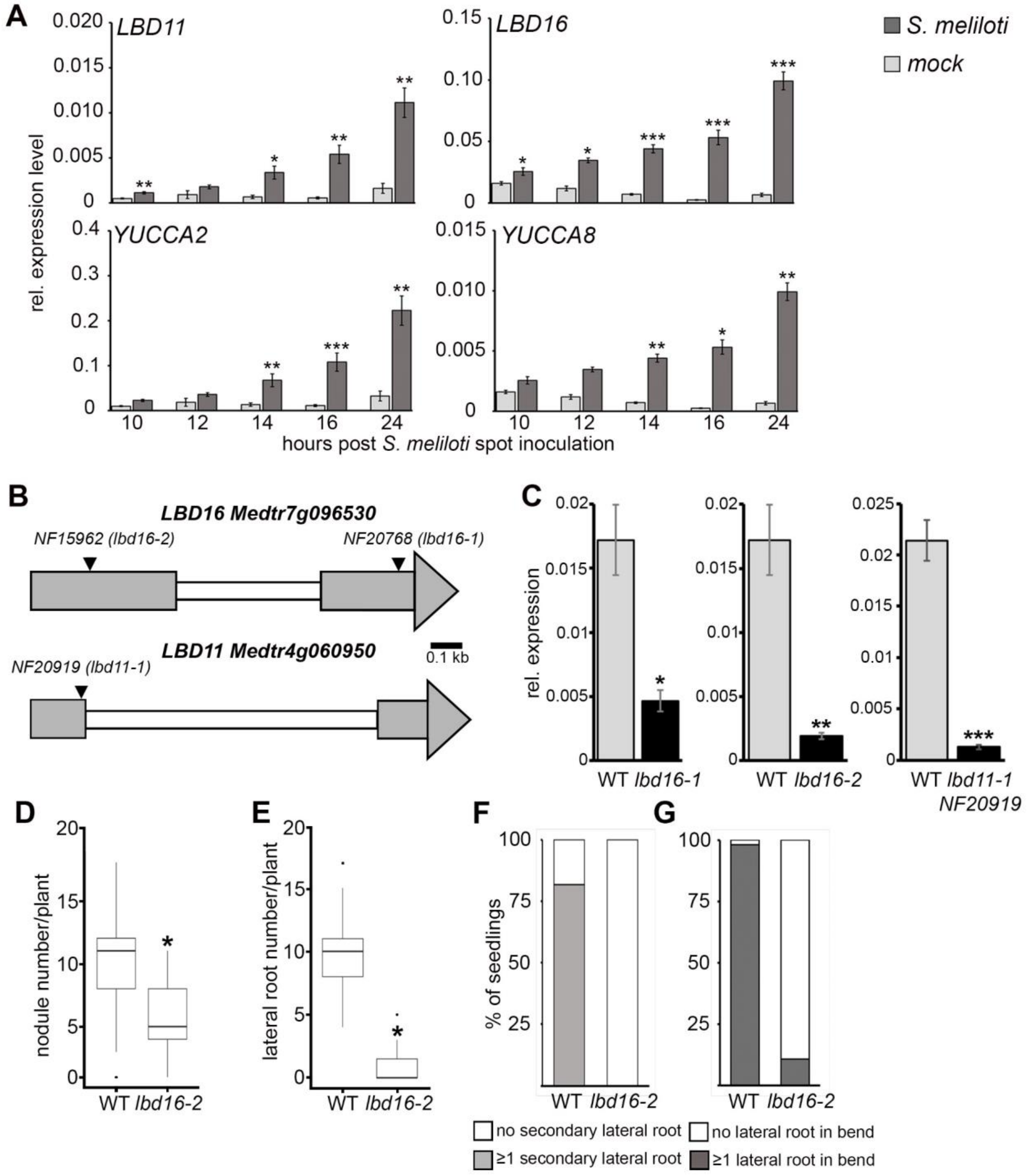

Figure S3. *LBD* and *YUC* genes are expressed during lateral root and nodule primordium initiation and development. Related to Figures 3 and 4. (A) Expression profiling on segments of *S. meliloti* and mock inoculated root sections. Expression levels were measured by qRT-PCR and normalized to *HH3*. Statistical comparisons were performed between *S.*

meliloti and mock inoculation. Values are the mean of 3 biological replicates ± SEM (Student’s t-test; * P < 0.05; ** P < 0.01, *** P < 0.001). **(B)** Location of exonic *Tnt1* retrotransposon insertions. **(C)** Comparison of transcript levels in WT and the corresponding exonic *Tnt1* retrotransposon insertion lines measured in 5-day old seedling roots. Expression levels were measured by qRT-PCR and normalized to *HH3*. Statistical comparison was performed between WT and the insertion lines. Values are the mean of 3 biological replicates (5 roots) ± SEM (Student’s t-test; * P < 0.05; ** P < 0.01, *** P < 0.001). **(D)** Distribution of nodule number per plant 14 days post *S. meliloti* spray inoculation of WT (n = 77) and *lbd16-2* (n = 89). Box plots show median (thick line), second to third quartiles (box), minimum and maximum ranges (lines), and outliers (single points). A one-way Kruskal-Wallis rank sum test showed that nodule number is dependent on genotype (KW = 91.649, df = 2, p = 2.2e-16). Asterisks indicate significantly different means for *lbd16-2* compared with WT, Dunn Test (95 % confidence). **(E)** Distribution of total lateral root number in 14-day old seedlings of WT and *lbd16-2.* Box plots as described in **(D)**. A one-way Kruskal-Wallis rank sum test showed that lateral root number is dependent on genotype. Asterisks indicate significantly different means for *lbd16-2* compared with WT. **(F)** Percentage of seedlings with ≥ 1 secondary lateral roots initiated from primary lateral roots in 14-day old seedlings, P-value 3.138e-21 (*lbd16-2*) compared to WT, Fisher’ s exact test. **(E-F)** WT (n = 49) and *lbd16-2* (n = 59). **(G)** Percentage of gravi-stimulated seedlings with ≥1 lateral roots in the bend 5 dpi, with P-value 1.005e-21 (*lbd16-2*) compared to WT, Fisher’s exact test, WT (n = 48) and *lbd16-2* (n = 56).

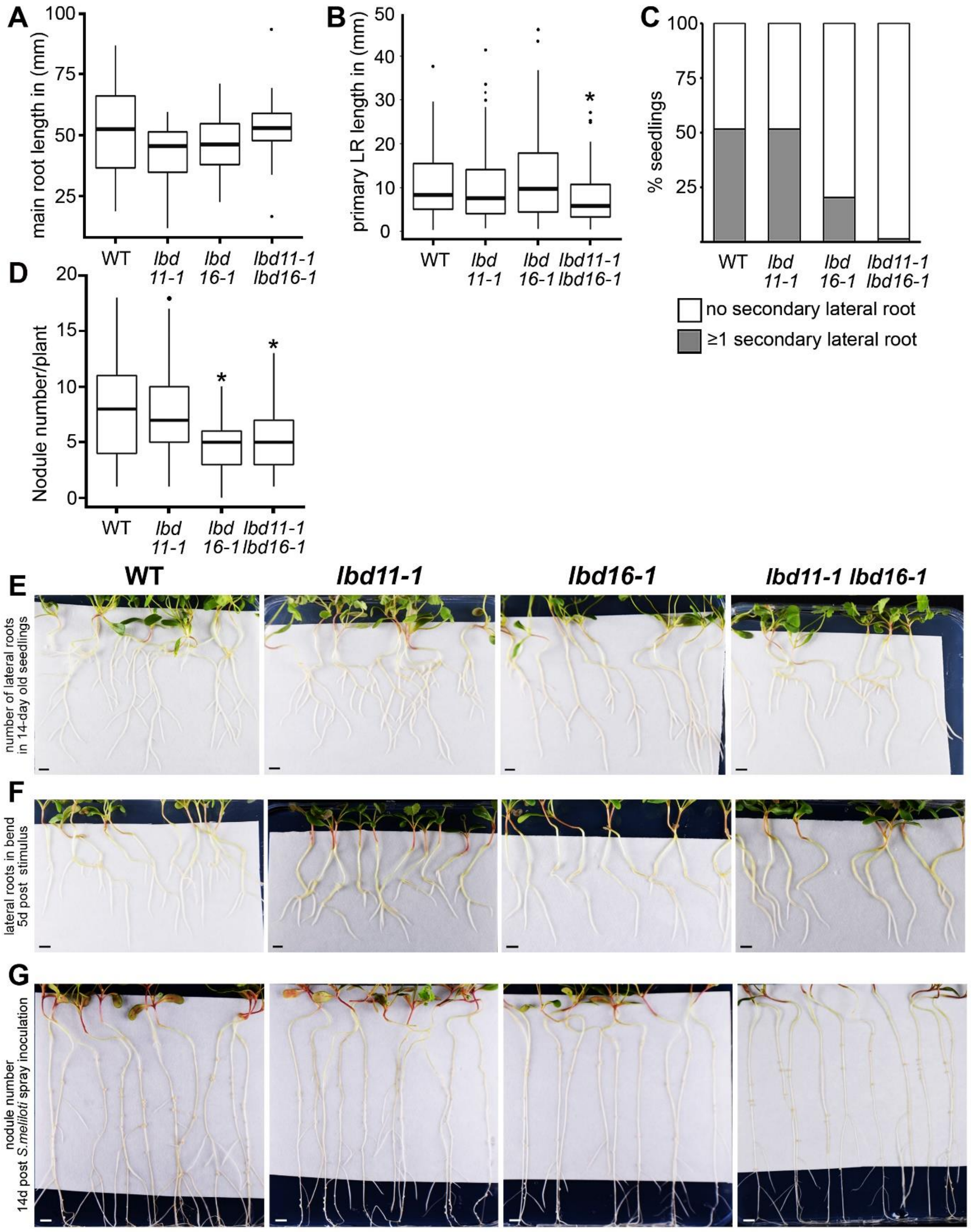

Figure S4. Lateral root and nodule number are reduced in *lbd16* but not *lbd11*. Related to Figure 4. (A-B) The primary root length was not significantly affected in *lbd11-1* and *lbd16-1*. Distribution of root length of the main roots **(A)** and primary lateral roots **(B)** of 14-day old seedlings of WT (n = 29), *lbd11-1* (n = 20), *lbd16-1* (n = 20), and *lbd11lbd16* (n = 29). Box plots

show median (thick line), second to third quartiles (box), minimum and maximum ranges (lines), and outliers (single points). A one-way ANOVA showed that the mean length of main roots and the primary lateral roots differed only slightly between genotypes. The asterisks indicate significantly different means for mutants compared with WT using Tukey multiple comparisons of means, 95% family-wise confidence level. * $P < 0.05$; ** $P < 0.01$, *** $P < 0.001$. **(C)** Percentage of seedlings with ≥1 secondary lateral roots initiated from primary lateral roots in 14-day old seedlings of WT (n = 56), *lbd11-1* (n = 58), *lbd16-1* (n = 64) and *lbd11lbd16* (n = 66) with P-values 0.571 (*lbd11-1*), 2.943e-04 (*lbd16-1*), and 1.650e-11 (*lbd11-1lbd16-1*), respectively, Fisher's exact test. **(D)** Distribution of nodule number per plant 14 days post *S. meliloti* spray inoculation of WT (n = 102), *lbd11-1* (n = 113), *lbd16-1* (n = 114) and *lbd11lbd16* (n = 94) seedlings. Box plots as described in **(A)**. A one-way Kruskal-Wallis rank sum test showed that nodule number is dependent on genotype (KW = 64.251, df = 3, p = 7.255e-14). Asterisks indicate significantly different means for *lbd16* and *lbd11lbd16* compared with WT, Dunn Test (95% confidence). **(E-G)** Images of WT, *lbd11-1*, *lbd16-1*, and *lbd11lbd16* plants grown on plates as used for the phenotyping assays (Figure 4 and Figures S3D-G and S4A-D). Total lateral root number, main root and primary lateral root length, and percentage of secondary lateral roots were assessed in 14-day old seedlings **(E)** percentage of plants with lateral roots induced in the bend was assessed 5 days post gravitropic stimulation **(F)**; nodule number was assessed in 16-day old plants 14 days post spray inoculation with *S. meliloti* **(G)**. Scalebars: 10 mm.

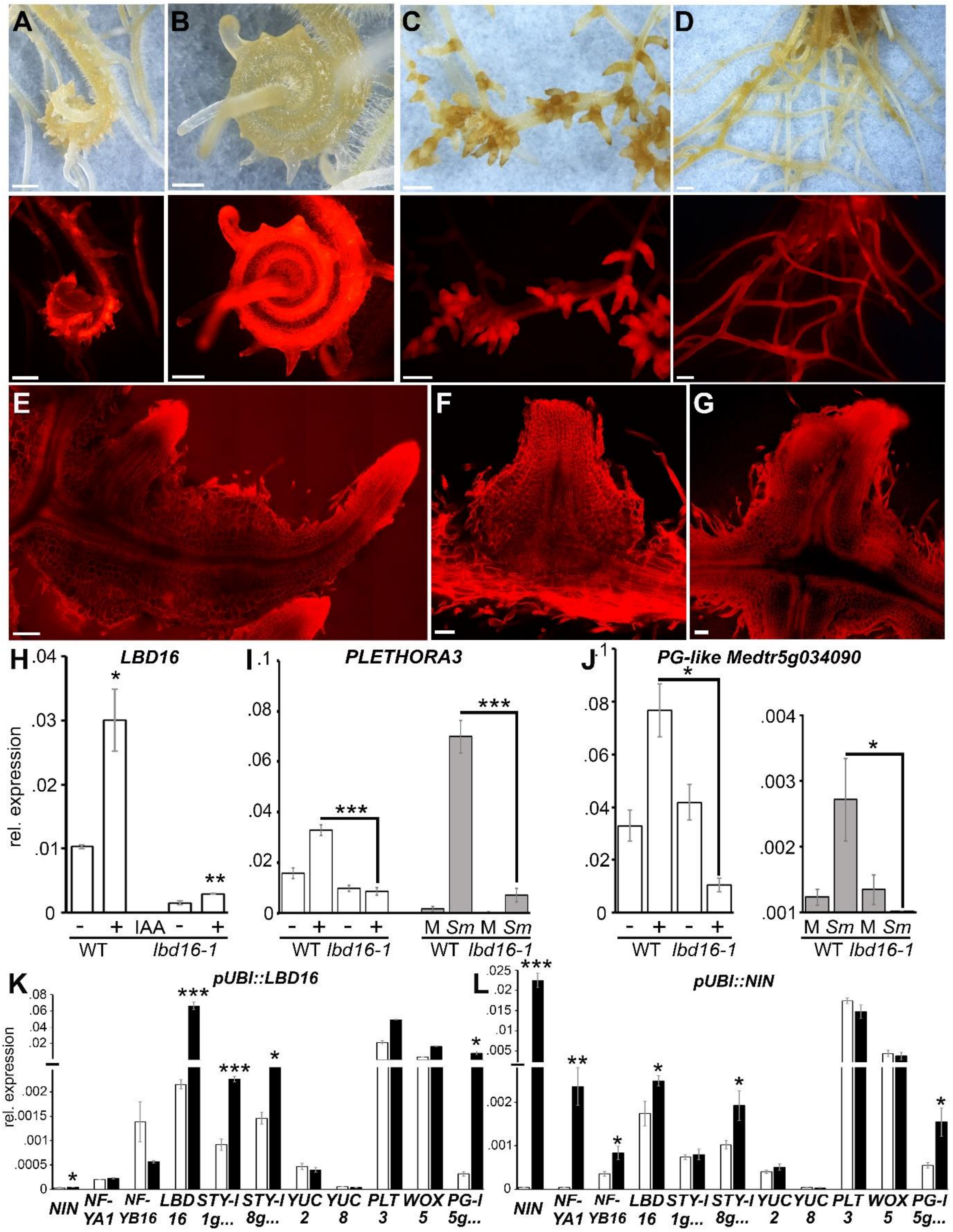

Figure S5. Constitutive expression of *LBD16* and *YUC2* results in ectopic primordium initiation. Related to Figures 5 and 6. (A-D) Hairy roots transformed with *pLjUBI::MtLBD16* **(A-B)** and *pLjUBI>GAL4UAS::MtYUC2* 3 weeks post dexamethasone **(C)** and mock **(D)**

treatment. **(A-D)** imaged in brightfield mode (upper panels) and with the transformation marker *pAtUBI:dsred* in epifluorescence mode (bottom panels). (**E-G)** Optical sections of PI stained root structures of *pLjUBI>GAL4UAS::MtYUC2* 3 weeks post dexamethasone treatment. Scale bars **(A-D)**: 1 mm, **(E-G)**: 50 µm. **(H)** Quantification of transcript levels of *LBD16* in WT and *lbd16-1* root sections 24 hrs post IAA (+) or mock (-) treatment. Expression levels were measured by qRT-PCR and normalized to *HH3*. Values are the mean of 3 biological replicates ± SEM (Student's t-test; Asterisks indicated statistical significance *, $P < 0.05$; **, $P < 0.01$, ***, $P < 0.001$). **(I-J)** Transcript levels of root organogenesis genes are affected by loss of *LBD16*. Gene expression levels of *PLETHORA3* **(I)** and *POLYGALACTURONASE*-like *(Medtr5g034090)* **(J)** in response to IAA (+) and mock (-) treatment (white bars), and *S. meliloti* and mock spot inoculation (grey bars) in *lbd16-1* and WT root sections (elongation-differentiation zone) at 24 hpi. Expression levels were measured by qRT-PCR and normalized to *HH3*. Statistical comparisons were performed between IAA and mock treatments, *S. meliloti* and mock inoculation, and between IAA treated and *S. meliloti* inoculated genotypes. Values are the mean of 3 biological replicates ± SEM (Student's t-test; Asterisks indicated statistical significance *, $P < 0.05$; **, $P < 0.01$, ***, $P < 0.001$). **(K-L)** Expression profiling on hairy root tissue constitutively expressing *LBD16* **(K)** and *NIN* **(L)** using the *L. japonicus UBIQUITIN* promoter. qRT-PCR normalized to *HH3*. Empty vector control (white bars)*;* overexpressing lines (black bars). Values are the mean of ≥3 biological replicates ± SEM (Student's t-test; * $P < 0.05$; ** $P < 0.01$, *** $P < 0.001$).

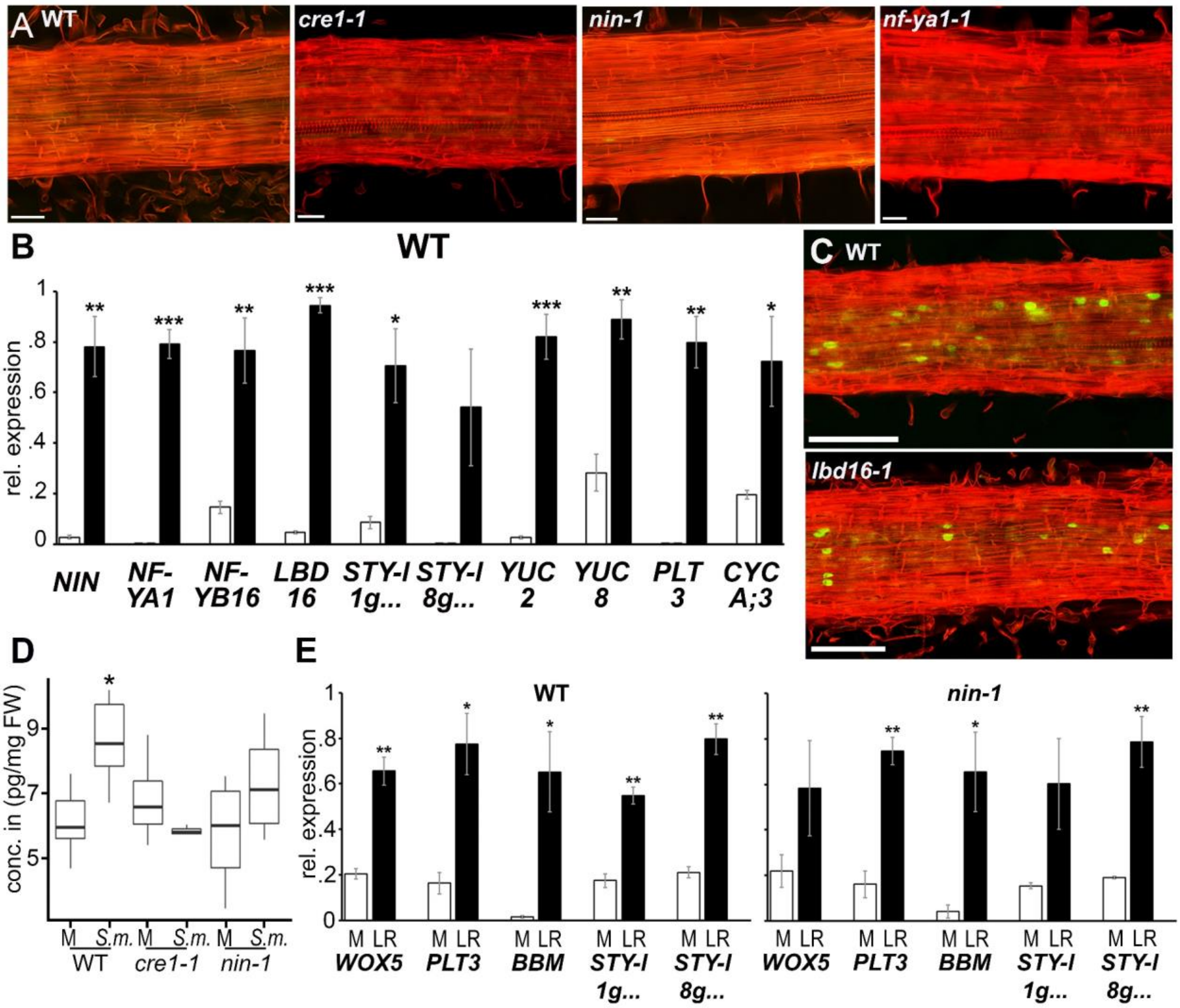

Figure S6. *lbd16* mediates the transcriptional response to auxin and cytokinin. Related to Figure 7. (A) Representative optical sections (≥ 5 roots analysed) through water-control treated WT*, cre1-1, nin-1* and *nf-ya1-1* segments of the primary root at the susceptibility zone. Cell walls were stained with PI and EdU labelling was used to detect DNA synthesis. No green labelled nuclei were observed under control treatments indicating that cell cycle activation was specifically induced by BAP treatment. Scalebars: 50 µm. **(B)** Transcript levels of selected genes (related to Figure 7A) measured by qRT-PCR on root segments of WT (ecotype *R108*) seedlings treated with cytokinin (24 hrs, 100 nM BAP). Expression levels were measured by qRT-PCR and normalized to *HH3*. Statistical comparisons were performed between hormone and mock treated roots. Values are the mean (Δ Ct values) of 3 biological replicates and the mean Δ Ct values of 3 biological replicates normalized to the maximum value within the dataset, ± SEM (Student's t-test; * $P < 0.05$; ** $P < 0.01$, *** $P < 0.001$). **(C)** Representative optical sections (≥10 roots analysed) through root segments (susceptibility zone) treated with

100 nM BAP of WT-R108 (top panel) and *lbd16-1* (bottom panel) for comparison (related to Figure 7). Red=cell walls, green nuclei = cell cycle activation. Scalebars: 100 µm. **(D)** IAA concentrations (pg/mg root freshweight) measured in root sections at 24 hrs post spot inoculation with *S. meliloti* (*S.m*) or mock (M). Asterisks indicate significant differences relative to mock, (Student's t-test; * $P < 0.05$; ** $P < 0.01$, *** $P < 0.001$), (n≥4, >200 plants/replicate). **(E)** Induction of lateral root associated genes is not affected in *nin-1* during lateral root initiation. Expression profiling on root segments 24 hrs post lateral root induction in 2-day old seedlings of WT and *nin-1*. Expression levels were measured by qRT-PCR and normalized to *HH3*. Statistical comparisons were performed between bent and unbent root sections. Values are the mean of 3 biological replicates ± SEM (Student's t-test; * $P < 0.05$; ** $P < 0.01$, *** $P < 0.001$) No significant difference in lateral root number between wild type and *nin-1* was observed with P-value 0.13, in n>45 plants per genotype, Fisher's exact test.

Name	Gene ID	Primer Sequence 5' to 3'	Primer Sequence 5' to 3'
**qRT-PCR**		**Forward**	**Reverse**
HH3	*Medtr4g097170*	CCCTGGAACTGTTGCTCTTC	CCTGAGCAATTTCACGAACC
NIN	*Medtr5g099060*	CTTTGCCGGAAGCCTAAAGGAC	TTTCAGAGTTGTAGGACACACACC
NF-YA1	*Medtr 1g056530*	ATCATCAGACGCAGGCATTCTCG	TCGTGCATATATGGCTTGTTACGC
NF-YB16	*Medtr4g119500*	ATGACAATGGCGGTATCAAGGAAC	TATCCGACCAACATTGGCTATTGG
LBD16	*Medtr7g096530*	AGCTCGTATCAGAGACCCTGTG	TGCAAGCATGCTACCTGTTGTTG
LBD11	*Medtr4g060950*	AGGCTAGTGCAAGAGTTAGAGACC	TGGAGTTGGCAAATTGCTCCTG
YUC2	*Medtr6g086870*	GGGTGTGGAAATTCAGGTATGGAG	AGGATGGATGAGCATTATGGTTGC
YUC8	*Medtr7g099330*	AGACTTTCTCTCACGCCGTTGC	ACCAGATGGACCTGCACCTATG
CycA3;1	*Medtr3g102530*	GCTTCTCCCTCAAACCCTTCA	CGATGAGCATGGATGAAACACC
PLT3	*Medtr5g031880*	CAAGCAAGAATTGGTCGTGTTGCC	TCTGCAGCTTCCTCTTCAGTTGCG
BBM	*Medtr7g080460*	TCACGAGGTGCATCCATTTACCG	TCTGCTGCCTCTTCTTGAGTGCTG
WOX5	*Medtr5g081990*	CTGGCACAAAGTGTGGTCGTTG	TTGATCAGTGCTTGGAGTTCTGAG
RR9	*Medtr3g015490*	TCCTCAGAGAATGTCCATCAAGG	TGTGGTTTCAGCTTGTTCACATC
RR11	*Medtr8g038620*	AGTAATGGGCATGGCAGCTGAG	AGGCCTTACTAGCAGAATCCACTG
RR19	*Medtr3g088630*	CCATTGCAGTTGCAAGAGGGAAAC	ATCCCAGGCATGCAATAATCTGTC
CKX-like	*Medtr4g126150*	TATCACGCGGTTCTTGGAGG	TTAACCGTTGTGGGAGCTGG
PG-like	*Medtr5g034090*	ACAGCAGCAAGTTAGCATGTGGAG	ATTCCATGTCCCGGACCACAGTTG
STY-like	*Medtr1g023230*	AGCAGCAGCAACAACAGTTTCAC	AAATTTCCCAACTCCAACCCTGTG
STY-like	*Medtr8g076620*	GGCGCACTTGTTGATCCTTC	ATTGCGTACCACTAGCCGTC
Genotyping			
Tnt1	*Tnt1 transposon*	TCCTTGTTGGATTGGTAGCC	CAGTGAACGAGCAGAACCTGTG
NF20768 lbd16-1	*Medtr7g096530*	GGGCCAGTCAAAGAATATTA	TTCGTCCTTGACACTCTCATT
NF15962 lbd16-2	*Medtr7g096530*	CCATAAAGAAATGTCTCCC	GAGAGACACAACCATACACAG
NF18998/20919 lbd11-1	*Medtr4g060950*	ACAAAGGGGAGTGTTATTAG	TCCTTGTTGGATTGGTAGCC

Table S1. Primers used in this study. Related to STAR methods.

L2 plasmids: GUS reporters
EC20325_pL2B-R1-pMedtr4g060950 (LBD11):GUS-t-LBD11-R2-pAtUBI:dsred-EC20325
EC20353_pL2B-R1-pMedtr7g096530(LBD16):GUS-t-LBD16-R2-pAtUBI:dsred-EC20253
EC21965_pL2B-R1-pAtUBI:KAN-R2-pMedtr6g086870(YUC2):GUS-t-35S-R3-pAtUBI:dsRed-EC21965
EC21966_pL2B-R1-pAtUBI:KAN-R2-pMedtr7g099330(YUC8):GUS-t-35S-R3-pAtUBI:dsRed-EC21966
**L2 plasmids: dexamethasone-inducible ectopic expression**
EC11480_pL2B-R1-pAtUBI:KAN-R2-p6xGAL4UAS:NLS-eGFP-pAtUBI:dsRed-R3-pLjUBI1:GVG-11480
EC21962_pL2B-R1-pMtGH3:GUS-R2-p6xGAL4UAS:Medtr6g086870(YUC2)-R3-pAtUBI:PM-mCherry-R4-pLjUBI1::GVG-21962
**L2 plasmids: ectopic expression using the *Lotus japonicus UBIQUITIN* promoter (*LjUBI*)**
EC52236_pL2B-R1-pLjUBI:Medtr7g096530(LBD16)-t35S-R2-pAtUBI:dsred-52236
EC52237_pL2B-R1-pLjUBI:Medtr4g060950(LBD11)-t35S-R2-AtUBI:dsred-52237
EC52357_pL2B-R1-AtUBI:dsred-R2-LjUBI:Medtr5g099060(NIN)-t35S-52357
EC52395 pL2B-R1-AtUBI:dsred-R2-LjUBI:t35S-52357
EC20681_pL2B-R1-pLjUBI:GFP-R2-pAtUBI:dsred-20681
EC11680_pL2B_R1-pAtUBI:KAN-R2-AtUBI:dsred-EC11680

Table S2. Constructs used in this study. Related to STAR methods.

SUPPLEMENTAL REFERENCE

S1. Moreno-Risueno, M.A., Van Norman, J.M., Moreno, A., Zhang, J., Ahnert, S.E., and Benfey, P.N. (2010). Oscillating Gene Expression Determines Competence for Periodic *Arabidopsis* Root Branching. Science *329*, 1306-1311.
