# Supplementary figures and images for: NODULE INCEPTION Recruits the Lateral Root Developmental Program for Symbiotic Nodule Organogenesis in Medicago truncatula
Source: Curr Biol. 2019 Nov 4;29(21):3657–3668.e5. doi: 10.1016/j.cub.2019.09.005 (PMC6839406; doi:10.1016/j.cub.2019.09.005)

A

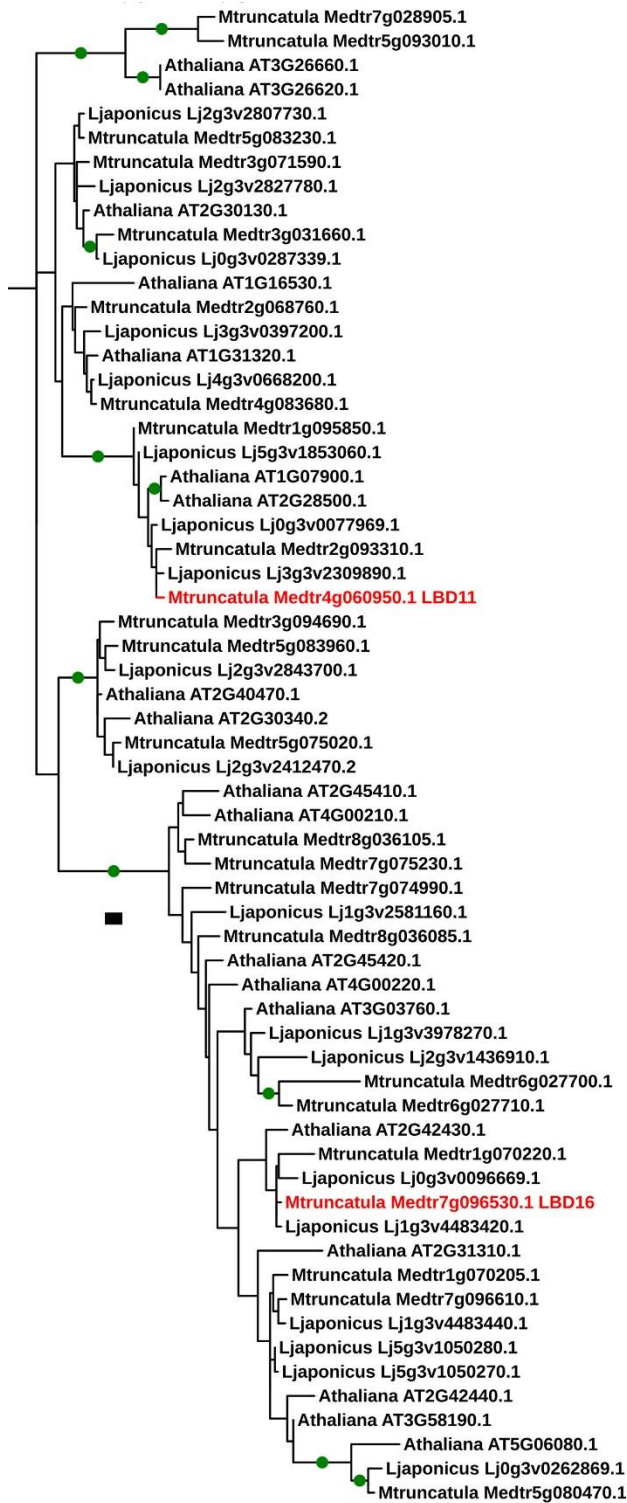

B

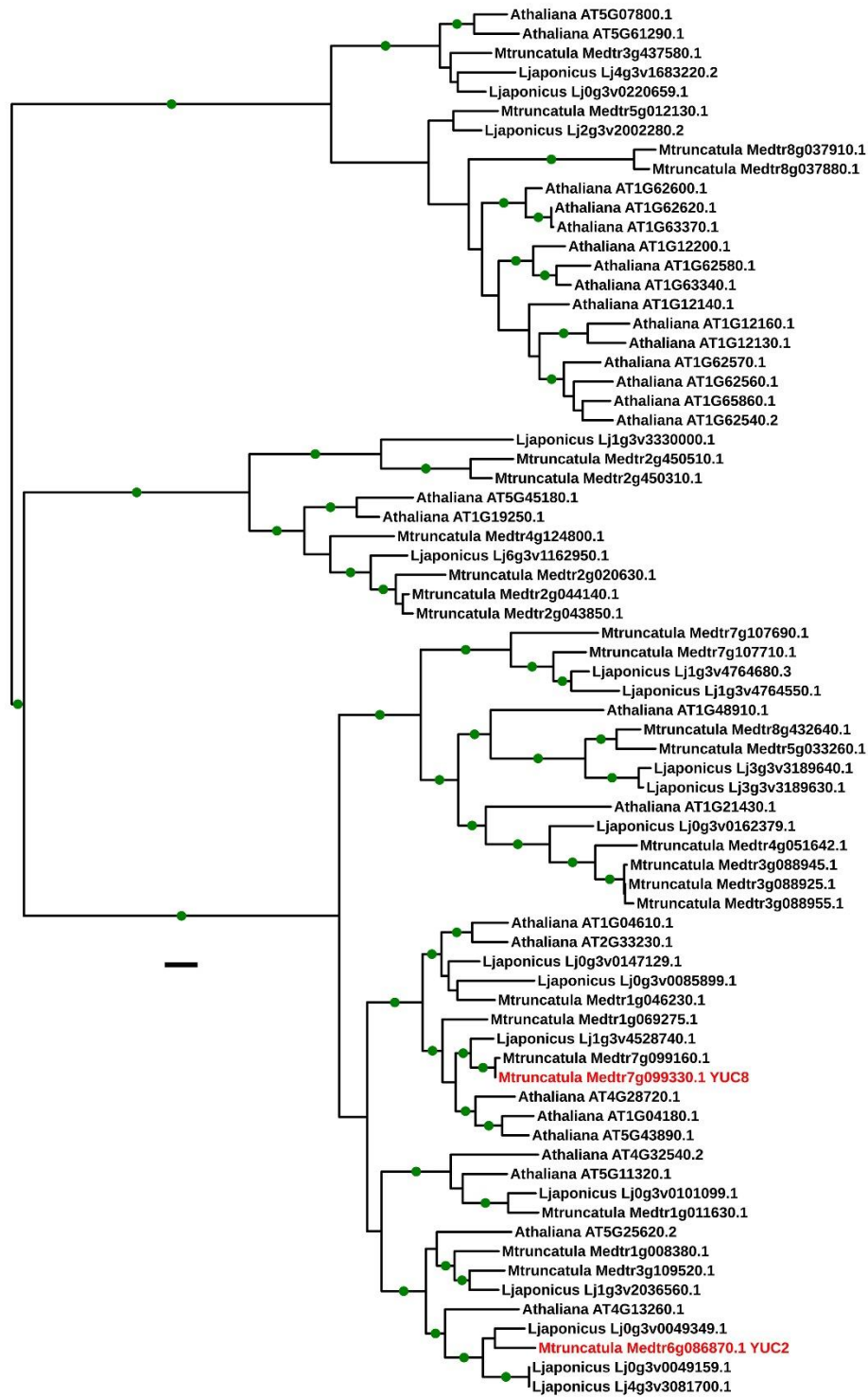

Supplement: Data S2. Phylogenetic Analysis, Related to Figures 3 and 4 and STAR Methods — (A) Phylogenetic analysis of the LBD gene family. The tree shows a clade of 62 proteins out of 139 LBD-like proteins from Arabidopsis thaliana, Lotus japonicus, and Medicago truncatula. (B) Phylogenetic analysis of the YUCCA gene family. The tree shows all YUCCA-like proteins identified from Arabidopsis thaliana, Lotus japonicus, and Medicago truncatula. Genes functionally characterized in this study are labeled in red. Green dots indicate bootstrap values > = 90%. Related to Figures 3 and 4. [file mmc3.pdf]
